# Identifying MicroRNAs and Transcript Targets in *Jatropha* Seeds

**DOI:** 10.1371/journal.pone.0083727

**Published:** 2014-02-13

**Authors:** Vanessa Galli, Frank Guzman, Luiz F. V. de Oliveira, Guilherme Loss-Morais, Ana P. Körbes, Sérgio D. A. Silva, Márcia M. A. N. Margis-Pinheiro, Rogério Margis

**Affiliations:** 1 Center of Biotechnology and PPGBCM, Laboratory of Genomes and Plant Populations, Federal University of Rio Grande do Sul - UFRGS, Porto Alegre, RS, Brazil; 2 Brazilian Agricultural Research – EMBRAPA, Pelotas, RS, Brazil; 3 PPGGBM at Federal University of Rio Grande do Sul - UFRGS, Porto Alegre, RS, Brazil; Duke-NUS, Singapore

## Abstract

MicroRNAs, or miRNAs, are endogenously encoded small RNAs that play a key role in diverse plant biological processes. *Jatropha curcas* L. has received significant attention as a potential oilseed crop for the production of renewable oil. Here, a sRNA library of mature seeds and three mRNA libraries from three different seed development stages were generated by deep sequencing to identify and characterize the miRNAs and pre-miRNAs of *J. curcas*. Computational analysis was used for the identification of 180 conserved miRNAs and 41 precursors (pre-miRNAs) as well as 16 novel pre-miRNAs. The predicted miRNA target genes are involved in a broad range of physiological functions, including cellular structure, nuclear function, translation, transport, hormone synthesis, defense**,** and lipid metabolism. Some pre-miRNA and miRNA targets vary in abundance between the three stages of seed development. A search for sequences that produce siRNA was performed, and the results indicated that *J. curcas* siRNAs play a role in nuclear functions, transport, catalytic processes and disease resistance. This study presents the first large scale identification of *J. curcas* miRNAs and their targets in mature seeds based on deep sequencing, and it contributes to a functional understanding of these miRNAs.

## Introduction

Increased energy consumption is a key concern of contemporary society as a result of rapidly diminishing fossil fuel deposits and increasing levels of carbon dioxide released into the atmosphere. Thus, environmentally friendly sources of fuels, such as bioethanol and biodiesel, are promising alternatives for fossil fuels. In this context, great interest has been generated regarding the potential of *Jatropha curcas* L. for biodiesel production. This species belongs to the Euphorbiaceae family and is found in almost all tropical areas; it occurs on a large scale in tropical and temperate regions [1], [2]. *J. curcas* has potential for biodiesel production because it is perennial, drought-resistant and has a high oil content (∼40%). Additionally, this crop can be grown in degraded soils (non-agricultural lands), which controls erosion without competing for food production habitats [3], [4].

In spite of having the potential for high fuel production, improving the quality of *J. curcas* seed oil remains challenging. The desired traits include increased oil content, decreased unsaturated fatty acid content (to increase the oxidative stability), decreased free fatty acid content (to prevent soap formation and increase biodiesel productivity) and decreased 18 carbon fatty acid content (to reduce viscosity) [5]. Furthermore, reducing seed toxicity and increasing pest tolerance are also desirable [1], [5].

MicroRNAs (miRNAs) are small non-coding RNAs that act as post-transcriptional regulators of gene expression [6]. They are typically transcribed by RNA Polymerase II as long polyadenylated transcripts, with an imperfect stem-loop structure known as pri-miRNA, which is recognized and processed by DICER-Like1 (DCL1) into an miRNA precursor (pre-miRNA). This sequence is further processed to generate mature miRNAs, which are composed of single-stranded RNA molecules of approximately 21 nucleotides (nt) in length [7]–[9]. To regulate protein-coding genes, the mature miRNA binds to sites in the 3′ and 5′ untranslated regions (UTR) or the coding sequence (CDS), leading to mRNA degradation or translational inhibition, depending on the degree of complementarity between the miRNA and its target transcript [8], [10]. This regulation plays critical roles in plant development and growth as well as a range of physiological processes, including abiotic and biotic stress responses [11], [12], and in lipid metabolism [13], [14].

At present, there have only been two recent studies regarding the identification of miRNAs from *J. curcas*. One study used a small RNA cloning methodology and did not provide the precursor sequence from Jatropha [15]. The other study used known plant miRNAs from Viridiplantae to search for *J. curcas* miRNAs in publicly available EST and GSS databases; therefore, only conserved miRNAs were identified by this approach [16]. In the present study, conserved and novel miRNAs from *J. curcas* were identified through the deep sequencing of small RNAs (sRNA) from mature seeds. The targets of these miRNAs were predicted *in silico*, and the results indicate that miRNAs are involved in a wide range of physiological processes in seeds, including growth, hormone signaling, stress resistance and lipid metabolism, among others. In addition, polyadenylated transcript sequencing (mRNA-seq) libraries from three seed development stages (immature, intermediate and mature) were used to identify and characterize the abundance of pre-miRNAs and miRNA targets, providing important information related to regulatory timing.

## Material and Methods

### *J. curcas* Seed Collection and RNA Isolation

For the RNA isolation, fruits from *J. curcas* plants grown in an open environment at Embrapa Clima Temperado (Pelotas, RS, Brazil) were collected at 10–20 (immature seeds), 20–40 (intermediate seeds) and 40–60 (mature seeds) days after flower opening (DAF). The seeds were dissected from their fruits and immediately frozen in liquid nitrogen and then stored at −80°C. Total RNA was isolated from a pool of seeds from each stage with Trizol (Invitrogen, CA, USA), according to the manufacturer’s protocol. RNA quality was evaluated by electrophoresis on a 1% agarose gel, and the RNA concentration was determined by Nanodrop (Nanodrop Technologies, Wilmington, DE, USA).

### Small RNA and mRNA-seq Library Construction using Deep Sequencing

Total RNA (>10 µg) was isolated from immature, intermediate and mature seeds and sent to Fasteris SA (Plan-les-Ouates, Switzerland) for processing and sequencing with an Illumina HiSeq2000. To generate the mRNA-seq libraries, polyadenylated transcript sequencing was performed as follows: Poly(A) mRNAs were purified, cDNA was synthesized using a Poly(T) primer shotgun to generate 500 nt inserts, 3p and 5p adapters were bound, and a cDNA colony template library was generated by PCR amplification, and 100 single-end bases were sequenced by Illumina sequencing. Illumina sequencing output data were sequence tags of 100 bases. Three mRNA-seq libraries were constructed from immature seeds (L1), from intermediate seeds (L2) and from mature seeds (L3).

Only RNA from mature seeds was used to produce the small RNA library. In brief, RNA bands corresponding to a size range of 20–30 nt were separated and purified from the acrylamide gel and subsequently bound to 3p and 5p adapters in two separate subsequent steps, each followed by acrylamide gel purification. Then, the cDNAs were synthesized and a cDNA colony template library was generated by PCR amplification for Illumina sequencing.

### Sequence Data Analysis

Figure S1 summarizes the overall data analyses performed with the sRNA library and mRNA-seq libraries. First, all low quality reads (FASTq value <13) were removed, and 5p and 3p adapter sequences were trimmed using the Genome Analyzer Pipeline (Fasteris). The remaining low quality reads with ‘n’ were removed using the PrinSeq script [17]. Sequences shorter than 18 nt and longer than 25 nt were excluded from further analysis. sRNAs derived from Viridiplantae rRNAs, tRNAs, snRNAs and snoRNAs (from the tRNAdb [18]; SILVA rRNA [19] and NONCODE v3.0 [20] databases); cpRNA from *J. curcas*; and mtRNA from *Ricinus communis* [from the NCBI GenBank database (http://ftp.ncbi.nlm.nih.gov)] were identified by mapping with Bowtie v 0.12.7 [21]. rRNAs, tRNAs, snRNAs and snoRNAs were excluded from the sRNA library that was used in further miRNA predictions and analyses.

The mRNA-seq libraries were first filtered for low quality “n” reads, and *J. curcas* coding sequence reads were obtained from the *Jatropha* genome database (http://www.kazusa.or.jp/jatropha). L1, L2 and L3 were pooled to produce mRNA contigs using the CLC Genome Workbench version 4.0.2 (CLCbio, Aarhus, Denmark) algorithm for *de novo* sequence assembly, with the default parameters (similarity = 0.8, length fraction = 0.5, insertion/deletion cost = 3, mismatch cost = 3), originating from the L1–L2–L3 library.

Sequence data from this article can be found in the GenBank data libraries under accession number(s) GSM1226039 (L1), GSM1226040 (L2), GSM1226038 (L3) and GSM1226041 (sRNA dataset).

### Prediction of Conserved and Novel miRNAs

To identify phylogenetically conserved miRNAs, reads from the sRNA library derived from mature seeds were mapped to a set of all mature Viridiplantae unique miRNAs obtained from the miRBase database (Release 19, August 2012) using Bowtie v 0.12.7 [21]. Only perfectly matched sequences were considered to be known miRNAs. To search for novel miRNAs, reads from the sRNA library derived from mature seeds were matched against contigs assembled from the L1–L2–L3 library using SOAP2 [22]. The SOAP2 output was filtered with an *in house* filter tool to separate pre-miRNA candidate sequences by using an anchoring pattern of one or two blocks of aligned sRNAs with a perfect match. MFOLD (http://mfold.bioinfo.rpi.edu/cgi-bin/rnaform1.cgi) was employed to predict hairpin structures with default parameters. Sequences were considered to be pre-miRNA if the RNA sequence could form an appropriate stem-loop structure with a mature miRNA sitting in one arm of the hairpin structure; the secondary structure had a high negative minimum folding free energy (MFE, 17–110 kcal/mol), using RNAstructure 5.3 [23], and a high negative minimum folding free energy index (MFEI, higher than 0.5). All putative pre-miRNAs were verified by a BLASTn algorithm from NCBI databases and the miRBase database (Release 19, August 2012). The frequency of identified miRNAs was obtained by aligning the conserved and novel precursors identified in this study and the sRNA library using Bowtie v 0.12.7, with the default parameters. The SAM files from Bowtie were then processed using *in house* Python scripts to count the frequencies of each read and map them into the three libraries. The most frequent miRNA for each precursor was designated as miRNA, while the others were designated as isomiRNAs.

### miRNA Targets Prediction

mRNA contigs from the L1–L2–L3 library were clustered by using Gene Indices clustering tools (http://compbio.dfci.harvard.edu/tgi/software/) [24] to reduce sequence redundancy. The clustering output was passed on to a CAP3 assembler [25] for multiple alignment and consensus building. Contigs that could not reach the threshold set and fell into a random assembly and remained as a list of singletons.

The predicted target genes of conserved and novel miRNAs was performed with a psRNAtarget [26] by aligning mature sequences against assembled *J. curcas* unigenes from the L1–L2–L3 library. Default parameters were used, and a maximum expectation of 4.0 was applied for the search with the most abundant miRNA. A maximum expectation of 5.0 was used to search the isomiRNA target genes, which were related to lipid metabolism pathways. An annotation of predicted targets was performed by using BLASTX from Blast2GO v2.3.5 software [27] based on their sequence similarity with previously identified and annotated genes from the NR and Swiss-Prot/Uniprot protein databases. The annotation was improved by analyzing conserved domains/families using the InterProScan tool, and Gene Ontology (GO) terms for the cellular component, molecular function and biological processes were determined by using the GOslim tool in the blast2GO software. Transcript orientations were obtained from the BLAST outputs.

### *In silico* Expression Analysis of Pre-miRNAs and miRNA Predicted Targets

To calculate the frequency of pre-miRNAs, mRNA reads from each individual library (L1, L2 and L3) were aligned in Bowtie v 0.12.7 by using the default parameters and allowing zero mismatches. For reference, all identified pre-miRNAs in this study were used. The SAM files from Bowtie were then processed, as discussed above. The scaling normalization method proposed by Robinson and Oshlack [28] was used for data normalization. Both R packages, EdgeR [29] and A–C test [30], were independently used to assess whether the pre-miRNA was differentially represented. In brief, EdgeR uses a negative binomial model to estimate the over dispersion from the pre-miRNA count. The dispersion parameter of each pre-miRNA was estimated by tagwise dispersion. The differential expression is then assessed for each pre-miRNA by using an adapted exact test for over dispersed data. The A-C test computes the probability that two independent counts of the same pre-miRNA came from similar samples. Pre-miRNAs were considered to be differentially represented if they had a p-value ≤0.001 in both statistical tests. The same method was adopted to evaluate the expression profile of predicted miRNAs targets, allowing two mismatches (one in the seed and another in the rest of the sequence).

### siRNA Prediction

siRNAs were identified by aligning *J. curcas* 24-nt sRNAs against the contigs from the L1–L2–L3 library. Putative contigs with a typical sRNA distribution pattern along the matching sequences [31] were further subjected to annotation using Blast2GO software, as described above.

## Results and Discussion

### Deep Sequencing of sRNAs and mRNA Libraries

To identify the conserved and novel miRNAs in *J. curcas* seeds, an sRNA library from mature seeds was constructed and sequenced by Illumina technology, resulting in a total of 16,771,931 reads. After removing the 3p and 5p adapter sequences and filtering out low quality “n” sequences, sRNAs within a 1–44 nt range were obtained, in which the majority were 18–26 nt in length (Table 1). Sequences shorter than 18 nt and longer than 25 nt were removed, resulting in 13,953,403 reads (Table 2), from which 5,400,278 reads were unique tags (38.7%). Approximately 80% (4,328,139) of the unique tags corresponded to singletons (Table S1).

**Table 1:** Raw data from sequencing *Jatropha curcas* sRNAs.

Read length (nt)	Number of reads	Percentage (%) of reads
0	12,163	0.073
1–17	1,347,333	8.330
18–26	14,175,504	84.190
27–44	516,355	3.790
Remaining	720,576	4.960

**Table 2:** Data from reads of the sRNA database of mature *Jatropha curcas* seeds filtered with non-coding RNAs.

Size	Total reads*	%	rRNA		tRNA		snRNA		snoRNA		cpRNA		mtRNA		all filters	
			totalreads	%	totalreads	%	totalreads	%	totalreads	%	totalreads	%	totalreads	%	totalreads	%
18	369,493	2.65	132,827	35.95	9,763	2.64	283	0.08	167	0.05	6,069	1.64	2,269	0.61	151,378	40.97
19	445,999	3.20	131,575	29.50	22,091	4.95	241	0.05	130	0.03	10,314	2.31	2,043	0.46	166,394	37.31
20	503,781	3.61	107,113	21.26	14,525	2.88	240	0.05	102	0.02	27,221	5.40	2,534	0.50	151,735	30.12
21	2,869,361	20.56	164,287	5.73	5,755	0.20	959	0.03	78	0.00	51,618	1.80	14,261	0.50	236,958	8.26
22	2,271,978	16.28	117,565	5.17	7,860	0.35	374	0.02	61	0.00	106,135	4.67	3,714	0.16	235,709	10.37
23	1,335,058	9.57	125,303	9.39	7,860	0.59	257	0.02	33	0.00	23,003	1.72	2,279	0.02	158,735	11.89
24	5,760,030	41.28	102,477	1.78	9,347	0.16	356	0.01	27	0.00	31,370	0.54	3,447	0.06	147,024	2.55
25	397,703	2.85	87,646	22.04	6,541	1.64	178	0.04	17	0.00	8,911	2.24	4,192	1.05	107,485	27.03
**Total**	13,953,403	100.00	968,793	6.94	83,742	0.60	2,888	0.02	615	0.00	264,641	1.90	34,739	0.25	1,355,418	9.71

*Total reads before filtering with non-coding and organellar small RNAs. The small RNAs were clustered according to their origin as follows: ribosome (rRNA), transporter (tRNA), small nuclear (snRNA), small nucleolar (snoRNA), mitochondrial (mtRNA) and chloroplastic (cpRNA).

Non-coding RNAs were also removed from the sRNA library for further analysis (Table 2). The sequence analysis showed that rRNA had the highest read frequency of all filtered sRNA classes, with 6.94% of the total reads. The majority of these rRNA sequences were found in the dataset with 21 nt-long sequences. Interestingly, 35.95% of the 18 nt sequences represented rRNAs. cpRNA sequences were the second most abundant filtered sequences after those from rRNA, corresponding to 1.9% of the total reads. tRNAs, mtRNAs, snRNAs and snoRNA were less frequent in the sRNA library. Taken together, 9.71% of the sRNA library was filtered with these RNA types, leaving 12,597,985 reads.

**Figure 1:**
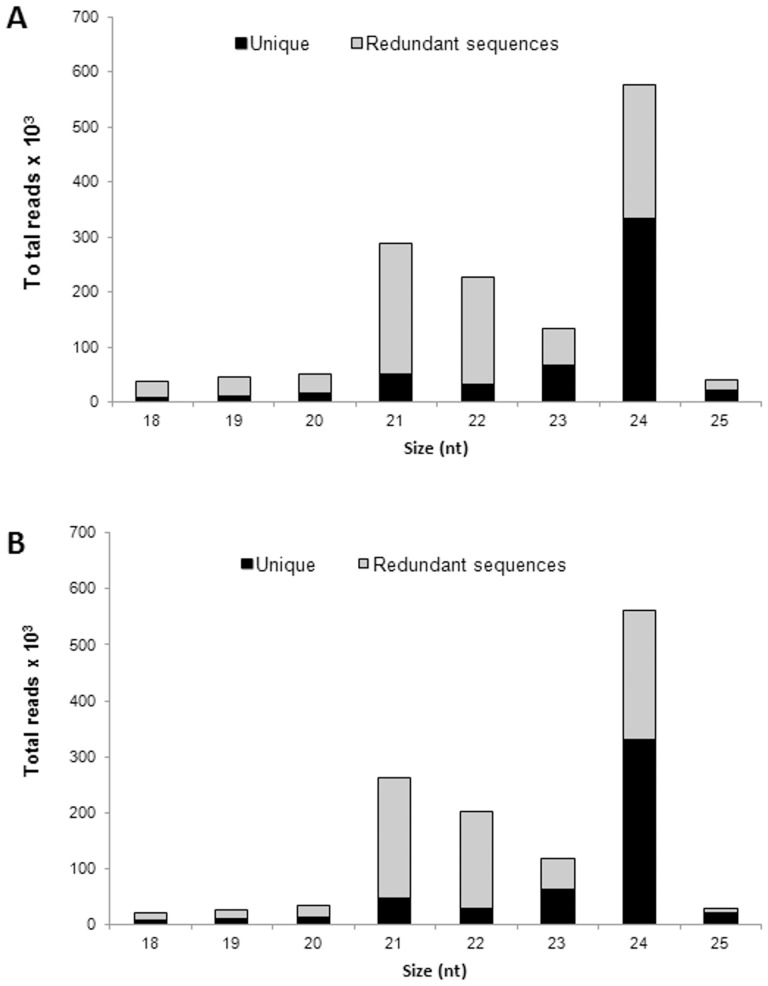
Total number of redundant and unique reads in the sRNA library of *J. curcas* mature seeds. (**A**) Data before filtering with non-coding RNAs and organelle RNAs. (**B**) Data after filtering with non-coding RNAs and organelle RNAs.

The length distribution of redundant and non-redundant sRNAs reads indicated that the most abundant and diverse sequences are within 21 (20.89%) and 24 nt (44.55%), a typical size range for Dicer-like (DCL)-derived products [9]. This distribution pattern for the small RNA size is similar to that of seeds from other species, such as Arabidopsis [32], peanut [13], barley [33], soybean [34] and canola [14], which implies that *J. curcas* possesses similar small RNA biogenesis processing components to other plant species. The same length distribution pattern was observed before and after filtering the sRNA library (Figure 1), indicating that the small RNA library was of high quality.

In this study, a specific mRNA transcriptome of *J. curcas* seeds was produced and used as a sequence reference for further analyses. Three mRNA seqs were obtained from seeds as follows: the L1 (immature seed) library yielded 43,328,830 reads, the L2 (intermediate mature) library yielded 35,062,185 reads, and the L3 (mature seed) library yielded 16,653,188 reads. All three libraries were pooled for *de novo* assembly (L1–L2–L3). The resulting 61,863 contigs had an average length of 755 bp, and the size ranged between 100 bp and 15,706 bp, with 27,520 contigs constituting more than 500 bp in length.

### Identification of Conserved miRNAs and Pre-miRNAs

**Figure 2:**
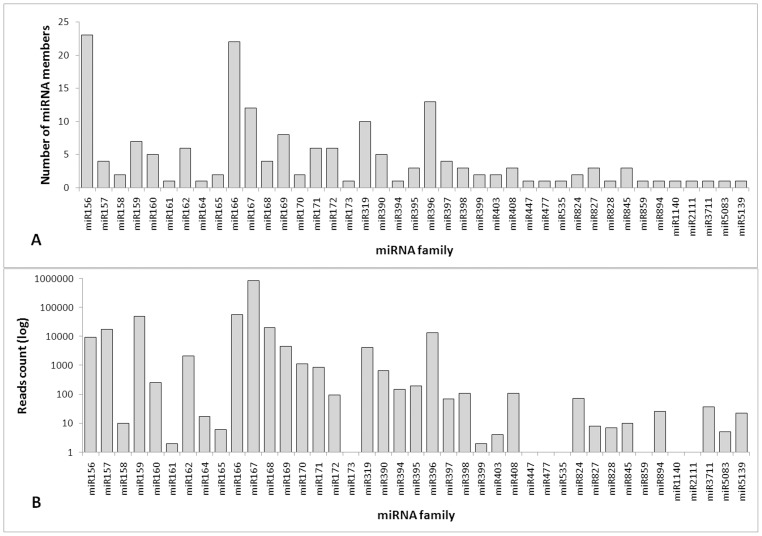
Known miRNA families identified in mature *J. curcas* seeds. (**A**) The total number of miRNA members (isomiRNAs) from each miRNA family. (**B**) The number of total read counts of each miRNA family.

Several studies have reported miRNA conservation across different plant taxa [35], [36]. To identify homologous miRNAs in the *J. curcas* sRNA library, a set of 2,585 unique mature plant miRNA sequences were extracted from the miRBase database and used for alignment against the sRNA library. Only sequences with exactly the same size and nucleotide composition were considered. A strict criterion of sharply defined distribution patterns for one or two block-like anchored sRNAs and at least 10 reads of a single miRNA sequence were used to predict novel miRNAs (see methods). The read depth distribution along putative pre-miRNAs was previously shown to be a reliable guide for differentiating possible miRNAs from contaminant sequences, such as the degradation products of mRNAs or transcripts that are simultaneously expressed in both sense and antisense orientations [14], [33], [37]. In total, 1,021,895 reads perfectly matched 177 conserved miRNAs belonging to 41 families, with an average of approximately 4 miRNA members per family (Table S2). Overall, the Jcu_MIR167 family was the most abundant conserved miRNA family present in *J. curcas* seeds, accounting for 842,066 reads, and the largest families were Jcu_MIR156 and Jcu_MIR166, with 23 and 21 members, respectively. Of the remaining miRNA families, 19 contained between 2 to 8 members, and 16 were represented by a single member (Figure 2 and Table S2). Furthermore, the results indicate that different members of the same miRNA family have clearly different expression levels (Table S2). For example, Jcu_MIR166 presents members ranging from 1 to 35,439 reads. Interestingly, the conserved miRNAs represented the most abundant *J. curcas* miRNAs and were distributed throughout seven families (MIR156, MIR157, MIR159, MIR166, MIR167, MIR168 and MIR396). These abundant miRNA families are largely found in Viridiplantae, indicating a fundamental role in plant life maintenance (Table S2).

In a study performed by Wang et al. [15], six conserved miRNAs were identified by the cloning approach from RNA libraries derived from theleaves and developing seeds of *J. curcas*. Two (Jcu_MIR166_3p and Jcu_MIR167_5p) of them were also identified in the present study, and three conserved miRNAs were identified only in the library from leaves. Moreover, the miRNA JcumiR004, which was identified as a novel plant miRNA, according to Wang et al. [15], was annotated in the present study as Jcu_MIR171_5p because the mature sequence showed a perfect match with the MIR171 from several species (Table S2, sequence UGAUUGAGCCGUGCCAAUAUC). Therefore, the discrepancies in identifying conserved miRNAs from the present study and the one performed by Wang et al. [15] correspond mostly to a difference in selected tissues and methods. During the cloning approach, there was a chance miRNAs with low expression level would not be detected. A more recent work by Vishwakarma and Jadeja [16] also focused on the identification of conserved miRNAs from *J. curcas* after transcript and partial genome sequence analysis. These authors were able to identify 24 predicted miRNAs belonging to five miRNA families (Jcu_MIR166, Jcu_MIR167, Jcu_MIR1096, Jcu_MIR5368 and Jcu_MIR5021). A lower number of miRNA families were identified by these authors relative to the present study, most likely because they used known plant miRNAs from Viridiplantae to search the conserved *J. curcas* miRNAs homologs in publicly available (and relatively small) EST and GSS databases compared to the database from the RNAseq generated in the present study.

To identify putative conserved pre-miRNA sequences, the sRNA library was matched against a set of *de novo* assembled contigs from three developmental stages of *J. curcas* seeds (L1–L2–L3 library). The candidate pre-miRNAs were predicted by exploring the secondary structure, the minimum folding free energy (MFE) and the minimum folding free energy index (MFEI). Candidate mRNA sequences with a stem-loop hairpin structure showing MFE values of 40–100 kcal/mol, MFEI values higher than 0.85 and more than 10 miRNA reads anchored in the same orientation in the 5p and/or 3p arm in a two block-like pattern were considered putative pre-miRNAs. The precursor identity was determined by BLAST searches against mature miRNAs in miRBase and the NCBI database. As a result, 41 known full-length plant pre-miRNA sequences were identified along with 18 miRNAs anchored in the 3p-arm and 19 miRNAs in the 5p-arm (Figure S2). The precursors had an average length of 154 bp, a CG content of 43.45%, an MFE of −53.13 and an MFEI of −0.86 (Table S3), which were similar to the pre-miRNA characteristics in other plant species [14], [35], [36]. Twenty conserved pre-miRNAs did not generate a hairpin structure according to MFOLD (http://mfold.bioinfo.rpi.edu/cgi-bin/rnaform1.cgi).

At the present, there is no miRNA sequence for *J. curcas* available in miRNA databases, and there is only one recently published report regarding the identification of miRNAs through the cloning of sRNA sequences, which did not provide the precursor sequence [15]. In this context, the use of deep sequencing technologies represents a powerful large scale approach for the reliable identification of miRNAs in *J. curcas* [14], [32], [33], [38], [39]. In the present study, the deep sequencing of an sRNA library from *J. curcas* mature seeds and three mRNAseq libraries from three stages of seed development allowed for the identification of conserved and species-specific miRNAs and pre-miRNAs.

### Identification of Novel miRNAs and Pre-miRNAs

In addition to conserved miRNAs, 16 sequences with characteristic hairpin-like structures were BLASTed against miRBase and NCBI databases, and no homologies with previously known plant miRNAs were found; these sequences characterize novel pre-miRNAs in plants. The identified pre-miRNAs had an average length of 162 bp and average MFE, MFEI and % CG content of −58.98, −0.96 and 41.50, respectively (Figure S3 and Table S4). Ten miRNAs were anchored in the 3p-arm and 15 miRNAs in the 5p-arm of these pre-miRNAs. The most abundant novel miRNA yielded 11,899 reads (Jcu_nMIR001), and it is the sixth most abundant miRNA in *J. curcas*, suggesting an important role in this tissue. The majority of novel miRNAs are 21 nt longer (Table S5), as was observed for conserved miRNAs. Interestingly, only one (JcumiR006) of the 46 novel miRNAs identified by Wang et al. [15] through cloning was identified in the present study (corresponding to JcuMIR0015_5p in the present study, sequence GGCAUGGGCGAUAUGGGCAAGA). This difference in the identified miRNAs is most likely a result of the chosen method, as explained earlier. Similarly, members of the Jcu_MIR166 and Jcu_MIR167 families demonstrated by Vishwakarma and Jadeja [16] were also identified in the present study. However, we were unable to identify six other members from the other families in our sRNA library, suggesting that these specific microRNAs may not be expressed or may not be detectable in mature seeds.

### Identification of IsomiRNAs

It was previously shown that miRNA variants, which are known as isomiRNAs, are detectable by high-throughput sequencing [40]–[43]. They show additional nucleotides in the 5′or 3′ terminus compared to the canonical or most abundant mature miRNAs. IsomiRNAs are considered to be a consequence of inaccuracies in Dicer pre-miRNA processing. Length heterogeneity can also arise by the exonuclease ‘nibbling’ of the ends, which produces a shorter template product, or by the post-transcriptional addition of one or more bases [44]. In the present study, the alignment of the sRNA library with identified *J. curcas* pre-miRNAs allowed for the estimation of the number and abundance of each isomiRNA corresponding to conserved (Table S2 and Figure S2) and novel *J. curcas* miRNA families (Figure S3 and Table S5).

IsomiRNAs were produced from both known and novel predicted pre-miRNAs of *J. curcas*. It was possible to observe that miRNA families differ significantly from each other in the number and abundance of isomiRNAs, as was observed in other studies [14], [34], [36]. The known pre-miRNA Jcu_MIR168 and the novel pre-miRNA Jcu_nMIR001 produced more isomiRNAs than the other pre-miRNAs. Unexpectedly, a variant of the novel Jcu_nMIR001 showed more than 10,000 reads because species-specific miRNAs usually present low levels of expression compared to the conserved miRNAs. The predicted targets of Jcu_nMIR001 miRNA are ribosomal proteins, which could explain the high abundance of this miRNA. It is also interesting to note that in some conserved miRNA families, the most abundant miRNA was not the canonical miRNA described for other species. This wide variation suggests that the same miRNA family is involved in divergent functions and may be necessary at different levels, according to the species, timing, tissue and/or other situations, such as environmental conditions and stresses. It has been shown that isomiRNAs can be expressed in a cell-specific manner, and numerous recent studies suggest that at least some isomiRNAs may affect target selection, miRNA stability, or loading into the RNA-induced silencing complex (RISC) [44].

### Abundance of *J. curcas* Pre-miRNAs during Seed Development

Because pre-miRNAs are processed as mRNAs species, *in silico* approaches can be used as a reliable tool for estimating the abundance of pre-miRNAs. Although this analysis does not directly predict the abundance of mature miRNA or the isomiRNAs, the number of precursor sequences present in seed developmental stages can provide information about the overall variation of the miRNA set generated from each precursor. The abundance analysis revealed that the majority of pre-miRNAs were present at similar levels in all libraries, suggesting that they are necessary during all seed development processes (Tables S3 and S4). Nevertheless, the abundance of some pre-miRNAs varied from one library to another, suggesting that the miRNAs from these precursors may be associated with the mRNA silencing involved in specific seed stage process, such as growth or maturation. Among the pre-miRNAs that were differentially represented, Jcu_MIR390 was only detected in L3 seeds; Jcu_MIR169a was only detected in L2 seeds; Jcu_MIR167a, Jcu_MIR172 and Jcu_MIR845 were only detected in L1 seeds (Table S3); Jcu_MIR156d, Jcu_MIR167b and Jcu_MIR319 were better represented in the L2 and L3 libraries; and Jcu_MIR390 was better represented in L3. Among the novel *J. curcas* miRNAs, Jcu_nMIR001 was better represented at the beginning of seed development, and Jcu_nMIR003, Jcu_nMIR007 and Jcu_nMIR016 were more represented in the intermediate seed stage (Table S4). It is also important to note that some novel pre-miRNAs were highly abundant, such as Jcu_nMIR001, which was the most expressed precursor during immature and mature seed stages, and Jcu_nMIR003, which was the most expressed in the intermediate stage, indicating that they play an important role in this plant. In summary, the prediction of pre-miRNA abundance among libraries increased the understanding and implications of post-transcriptional regulation in *J. curcas* seeds.

### siRNA in *J. curcas* Seeds

In contrast to the biogenesis and action of miRNA, siRNAs are 24-nt long sequences produced by double-stranded RNAs that can act at the transcriptional level through DNA methylation and histone modification and at the post-transcriptional level through the regulation of gene expression [45]. Several siRNAs have been recognized to play important roles in plant stress tolerance [46], [47]. Because of the large abundance of 24-nt sequences in the *J. curcas* sRNA library, we investigated the presence of siRNAs. The sRNAs that were 24 nt were matched against contigs assembled from L1–L2–L3 libraries. Putative contigs with typical sRNA distribution patterns along the matching sequences were further subjected to annotation (see methods). As a result, 42 siRNA precursors were identified and annotated (Table S6). This analysis indicates that siRNAs play a role in nuclear functions and in transport, catalytic processes and disease resistance. As expected, transposons and retroelements were relatively abundant among siRNA precursors, supporting their mechanism of action in guide chromatin-based events and resulting in transcriptional silencing [48]. It was reported that siRNA precursors can also be formed by cellular RNA-dependent RNA polymerase activity (RdRp) [49]. In fact, most *J. curcas* siRNA precursors were annotated as RdRps. Out of these precursors, the majority are associated with nuclear functions and play roles in transport, catalytic processes and disease resistance.

### Prediction of *J. curcas* miRNA Targets

Because the roles of miRNAs during plant development and in species-specific adaptation processes are executed through the cleavage or translation repression of target genes [6], miRNA target prediction is critical for gaining insight into the regulatory functions of miRNAs. In this study, the putative target genes of *J. curcas* miRNAs were predicted by using the web-based computer server psRNATarget, based on perfect or near perfect complementarity between miRNAs and their targets. The most abundant mature miRNAs from each family (isomiRNAs were not considered) were aligned with a set of unigenes generated from assembled *J. curcas* contigs from all seed development mRNA-seq libraries (L1–L2–L3). A total of 57 sequences were predicted as potential targets of 28 known plant miRNAs and 12 novel miRNAs, with an average of 1.8 targets per miRNA (Table S7).

**Figure 3:**
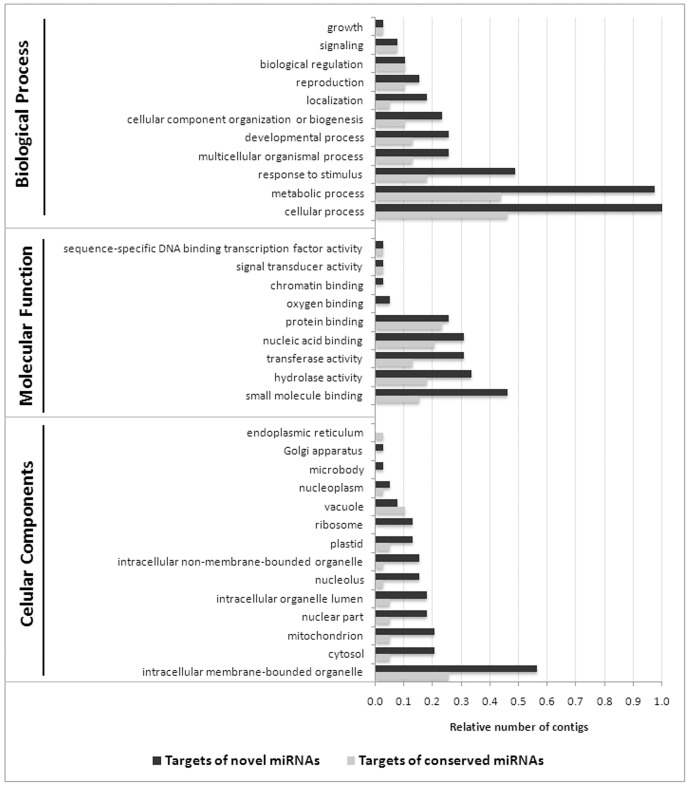
Targets of the miRNAs identified in mature seeds of *J. curcas*. The percentage (%) of contigs for each Gene Ontology (GO) term is relative to the total number of contigs from each gene category.

All of the identified targets were analyzed by using BLASTX against protein databases, followed by a GO analysis to evaluate their putative functions. According to the categorized GO annotation, 109 genes are involved in cellular components, with the majority of conserved and novel miRNA targets localized in intracellular membrane-bounded organelles. In the molecular functions category, 107 genes participate in catalytic or signaling transduction activities and binding activities with proteins and nucleic acids (Figure 3). With respect to biological processes, 216 genes primarily participate in stimulus responses and different cellular and metabolic processes, suggesting that the novel and conserved *J. curcas* miRNAs are involved in a broad range of physiological functions. These functions include participation in plant growth and development (pentatricopeptide repeat-containing protein, auxin response factor 10, seed maturation protein, etc.), lipid metabolism (phosphatidylserine decarboxylase and glycerophosphoryl diester), nutrient/cellular transport (amino acid transporter, high affinity nitrate transporter, metal transporter nramp6-like, etc.), and, primarily, in defense (tir-nbs-lrr resistance protein, cc-nbs-lrr resistance protein, cytosolic class I small heat shock protein partial, proline synthetase associated, dehydration responsive element binding proteins, etc.) (Table S7). The miRNAs targets were differentially represented during seed development, suggesting that they are regulated according to seed needs. For example, caffeoyl-o-methyltransferase and seed maturation protein are the most represented targets at the end of seed development, and subtilisin-like protease-like and auxin response factor 10 are better represented at the beginning of seed development than the other targets.

The auxin response factor (ARF) is a plant-specific family of DNA binding proteins involved in hormone signal transduction [50], [51]. The ARF gene and the F-box family proteins, also previously described in relation to auxin signaling [52], were predicted targets of conserved *J. curcas* miRNAs and were highly abundant in immature seeds. These genes, as well as some other predicted targets, such as proteins associated with nucleotide synthesis, ribosomal proteins, metal transporters and membrane proteins, may play a role in seed growth and formation. Another important gene targeted by Jcu_MIR156, Jcu_MIR168, Jcu_MIR403, Jcu_MIR472 and the novel Jcu_nMIR005 and Jcu_nMIR009 is the pentatricopeptide repeat gene (PPR), which participates in the regulation of gene expression and was present at considerable levels in all seed developmental stages, especially immature *J. curcas* seeds (Table S7). PPR belongs to a large gene family implicated in post-transcriptional processes, such as splicing, editing, processing and translation, in specific organelles, such as mitochondria and chloroplasts [53].

Several predicted targets of *J. curcas* miRNAs are involved in abiotic stresses, including genes associated with proline and phenylpropanoid synthesis, hormones and responses to dehydration and high temperatures. The dehydration-responsive element/C-repeat (DRE/CRT) was predicted as the target of Jcu_MIR156 and Jcu_MIR168. DRE/CRT has been identified as a cis-acting element involved in one of the ABA independent regulatory systems of abiotic stress response. In Arabidopsis, MIR156 and MIR168 were described as dehydration stress-responsive miRNAs [54]. An analysis of *J. curcas* DREB gene expression was performed by Tang et al. [55], and the expression was induced by cold, salt and drought stresses. Although only a few reads corresponding to this gene were detected in the present study, it could be interesting to investigate the role of miRNAs in the regulation of DREB expression in other tissues and during abiotic stresses, especially in *J. curcas* plants that are well-known for their tolerance to drought stress [56].

In addition to abiotic stress, miRNAs from *J. curcas* seeds may also participate in biotic stresses. The recognition of an invading microbial pathogen is often followed by the synthesis of specific plant disease resistance proteins (R) [57] that possess activities that can lead to plant cell death through the familiar hypersensitive response (HR), a characteristic feature of many plant defense mechanisms [58]. Genes encoding R proteins (cc-nbs-lrr resistance protein, disease resistance rpp13-like protein 1-like and tir-nbs-lrr resistance protein) and an HR protein were the predicted targets of Jcu_MIR159 and Jcu_MIR472 and were highly abundant at the end of seed development (Table S7), most likely as a mechanism to protect the mature seed against pathogens, thereby preventing unsuccessfully germination.

### Prediction of *J. curcas* miRNA Targets Involved in Lipid Metabolism

*Jatropha* has emerged as a promising biodiesel crop, but increasing the oil content and improving the oil quality are still challenging [2]–[4]. To investigate the involvement of isomiRNAs in regulating the lipid metabolism of *J. curcas*, the putative target genes of isomiRNAs with more than 10 reads were searched. This approach allowed for the identification of 12 miRNA targets related to lipid metabolic pathways (Table S8). This is the first report of miRNA associations with genes from the lipid metabolism in *J. curcas*. When estimating the sequence abundance of the target contigs, only hydroxysteroid 11-beta-dehydrogenase 1-like protein was better represented at the end of seed development, and phosphatidylserine decarboxylase, diphosphomevalonate decarboxylase, 1-phosphatidylinositol-bisphosphate, lipid binding, phosphatidylserine synthase 2 and cyclopropane-fatty-acyl-phospholipid synthase were more represented at the beginning of seed formation.

LPAT, or 1-acyl-sn-glycerol-3-phosphate acyltransferase, was the predicted target of Jcu_MIR001 and Jcu_MIR007 isomiRNA. This enzyme provides the key intermediate in membrane phospholipid and storage lipid biosynthesis in developing seeds [59]. Through the analysis of miRNA target abundance, it was possible to observe that LPAT was more represented in intermediate seeds. It was proposed that increasing the LPAT expression in seeds leads to a greater flux of intermediates through the Kennedy pathway and enhanced triacylglycerol accumulation [43]. Glycerophosphoryl diester (predicted target of Jcu_MIR001) and glycerol-3-phosphate dehydrogenase (predicted target of Jcu_MIR403) provide glycerol-3-phosphate for TAG assembly in the Kennedy pathway, and they were also more represented in intermediate seeds. These results confirm the general observation that storage lipid biosynthesis usually occurred at the middle-late stage during seed development [60] and suggest that the seed already reached maturity by the intermediate stage. Interestingly, a seed maturation protein target of Jcu_MIR472 was better represented in the intermediate stage, corroborating this finding. The same LPAT and GPDH expression pattern during seed development was observed by Xu et al. [61] and Guo et al. [43] by using real time PCR, which confirmed these results. In the study by Xu et al. [61], the authors verified that the gene encoding fatty acid desaturase exhibited low expression in all seed developmental stages, as was observed in the present study, suggesting that the transcript accumulation of this gene is not necessarily linked to triacylglycerol biosynthesis in developing *Jatropha* seeds.

## Conclusions

There is some uncertainty about the genetic contribution to divergent phenotypes and the response to growth conditions and stress in *Jatropha* genotypes because there are several reports indicating low genetic diversity among these plants. Epigenetic polymorphisms were suggested to be involved [62] but may not explain all of the observed the variation. The present study identified the *J. curcas* miRNAs that are involved in a wide range of physiological functions and therefore may also contribute to this phenotypic variation. The identification of a large set of miRNAs and their targets as well as siRNAs in *J. curcas* seeds contributes to the elucidation of complex miRNA-mediated regulatory systems, which control seed development and other physiological processes. Several *Jatropha* miRNAs were predicted to regulate genes associated with lipid metabolism; these miRNAs are promising candidates for improving the yield and quality of *J. curcas* seed oil. However, further studies are necessary to search for more novel miRNAs and to validate their target by expression analysis during seed development and also under specific environmental and physiological conditions.

## Supporting Information

Figure S1 **Flow chart of the methodology adopted to identify** ***J. curcas*** **miRNAs.**
(PDF)

Figure S2 **Predicted secondary structures of known miRNA precursors in** ***J. curcas*** **.** Locations and expressions of small RNAs mapped onto these precursors are presented. Read sequences corresponding to miRNA candidates, which are located in the 5p and 3p arms and labeled in red and purple, respectively. Values on the left side of the miRNA sequences represent the miRNA length (Jn) and read counts (x n) in the mature seed library.
(PDF)

Figure S3 **Predicted secondary structures of novel miRNA precursors in** ***J. curcas*** **.** The locations and the expression of small RNAs mapped onto these precursors are shown here. The sequences of miRNA candidates located in the 5p and 3p arms are labeled in red and purple, respectively. Values on the left side of the miRNA sequence represent miRNA length (Jn) and read counts (x n) in the mature seed library.
(PDF)

Table S1 Abundance of small RNAs from each size according to the number of reads.
(XLSX)

Table S2 Known miRNAs identified in the *J. curcas* sRNA library. This identification was performed by selecting perfect homologies to plant-conserved miRNAs, as deposited in the miRBase database (release 19, August 2012).
(XLSX)

Table S3 Putative known miRNA precursors in *J. curcas* and their abundance during seed development.
(XLSX)

Table S4 Putative new *J. curcas* miRNA precursors and their abundance during seed development.
(XLSX)

Table S5 Novel miRNAs identified in the sRNA library of *J. curcas*.
(XLSX)

Table S6 Putative precursors of siRNA in *J. curcas*.
(XLSX)

Table S7 Predicted targets of known and new miRNAs in mature *J. curcas* seeds. The abundance of these targets during seed development is shown here.
(XLSX)

Table S8 Predicted targets of known and new isomiRNAs related to the lipid metabolism of *J. curcas*. The abundance of targets during seed development is shown here.
(XLSX)
