# Supplementary figures and images for: Identifying MicroRNAs and Transcript Targets in Jatropha Seeds
Source: PLoS One. 2014 Feb 13;9(2):e83727. doi: 10.1371/journal.pone.0083727 (PMC3923737; doi:10.1371/journal.pone.0083727)

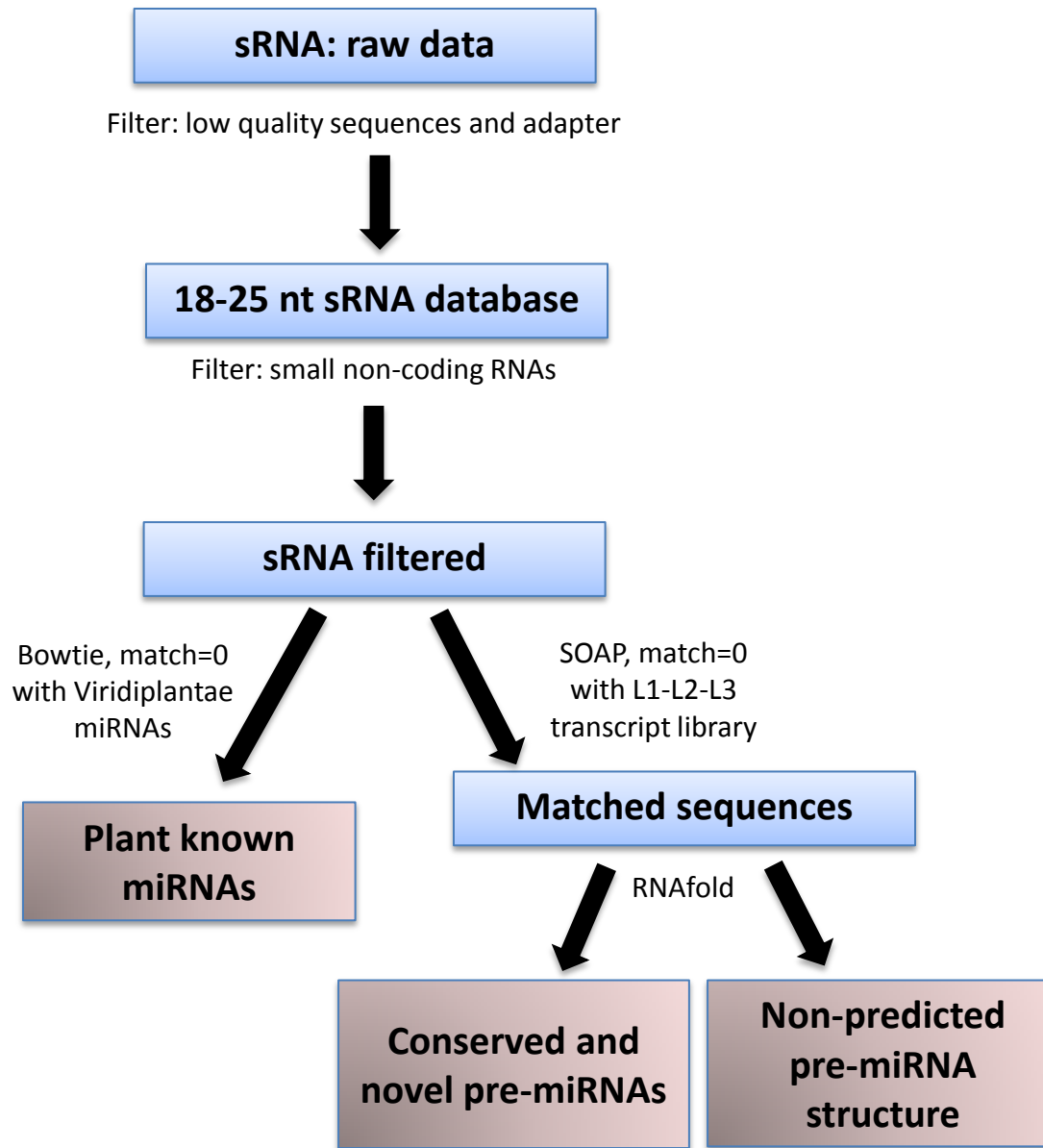

**Figure S1.** Flow chart of the methodology adopted to identify *J. curcas* miRNAs.

Supplement: Figure S1 — Flow chart of the methodology adopted to identify J. curcas miRNAs. (PDF) [file pone.0083727.s001.pdf]
