# Supplementary material for: Identifying MicroRNAs and Transcript Targets in Jatropha Seeds
Source: PLoS One. 2014 Feb 13;9(2):e83727. doi: 10.1371/journal.pone.0083727 (PMC3923737; doi:10.1371/journal.pone.0083727)

Figure S2. Predicted secondary structures of known miRNA precursors in *J. curcas*. Locations and expressions of small RNAs mapped onto these precursors are presented. Read sequences corresponding to miRNA candidates, which are located in the 5p and 3p arms and labeled in red and purple, respectively. Values on the left side of the miRNA sequences represent the miRNA length (Jn) and read counts (x n) in the mature seed library

Jcu_MIR157

```
 1 Jcu_miRNA157       100.0%  ATTTTGTTGACAGAAGATAGAGAGCACTGATGATGATATGCAAAATTAATCAATTAATGGGCTCTCTCTCTTTCTCTGCATTTCACTCTTCTGTGCTCTCTATGCTTCTGTCATCACCT
 2 J22_1751527_x8     100.0%  ----------------------------------------------------------------------------------------TTCTGTGCTCTCTATGCTTCTG---------
 3 J20_191301_x39     100.0%  ----------------------------------------------------------------------------------------------GCTCTCTATGCTTCTGTCAT-----
 4 J21_1529060_x1069  100.0%  ----------------------------------------------------------------------------------------------GCTCTCTATGCTTCTGTCATC----
 5 J22_1636813_x27    100.0%  ----------------------------------------------------------------------------------------------GCTCTCTATGCTTCTGTCATCA---
 6 J21_2419849_x1     100.0%  -----------------------------------------------------------------------------------------------CTCTCTATGCTTCTGTCATCA---
 7 J20_216262_x6      100.0%  -----------------------------------------------------------------------------------------------CTCTCTATGCTTCTGTCATC----
 8 J19_161765_x60     100.0%  ----------------------------------------------------------------------------------------------GCTCTCTATGCTTCTGTCA------
 9 J19_193920_x2      100.0%  ------------------------------------------------------------------------------------------------TCTCTATGCTTCTGTCATC----
10 J18_145130_x3      100.0%  -----------------------------------------------------------------------------------------------CTCTCTATGCTTCTGTCA------
11 J18_146021_x3      100.0%  ----------------------------------------------------------------------------------------------GCTCTCTATGCTTCTGTC-------
12 J18_202918_x1      100.0%  -------------------------------------------------------------------------------------------------CTCTATGCTTCTGTCATC----
13 J20_329851_x1      100.0%  ----TGTTGACAGAAGATAGAGAG-----------------------------------------------------------------------------------------------
14 J21_1929132_x39    100.0%  ----TGTTGACAGAAGATAGAGAGC----------------------------------------------------------------------------------------------
15 J19_178779_x7      100.0%  ------TTGACAGAAGATAGAGAGC----------------------------------------------------------------------------------------------
16 J20_170276_x144    100.0%  ------TTGACAGAAGATAGAGAGCA---------------------------------------------------------------------------------------------
17 J21_1228639_x4732  100.0%  ------TTGACAGAAGATAGAGAGCAC--------------------------------------------------------------------------------------------
18 J22_1356637_x351   100.0%  ------TTGACAGAAGATAGAGAGCACT-------------------------------------------------------------------------------------------
19 J21_2080381_x7     100.0%  -------TGACAGAAGATAGAGAGCACT-------------------------------------------------------------------------------------------
20 J20_294741_x1      100.0%  --------GACAGAAGATAGAGAGCACT-------------------------------------------------------------------------------------------
21 J20_184868_x60     100.0%  -------TGACAGAAGATAGAGAGCAC--------------------------------------------------------------------------------------------
22 J19_173200_x15     100.0%  -------TGACAGAAGATAGAGAGCA---------------------------------------------------------------------------------------------
23 J18_128682_x44     100.0%  --------GACAGAAGATAGAGAGCA---------------------------------------------------------------------------------------------
24 J19_184532_x3      100.0%  --------GACAGAAGATAGAGAGCAC--------------------------------------------------------------------------------------------
25 J18_138885_x8      100.0%  ------TTGACAGAAGATAGAGAG-----------------------------------------------------------------------------------------------
```

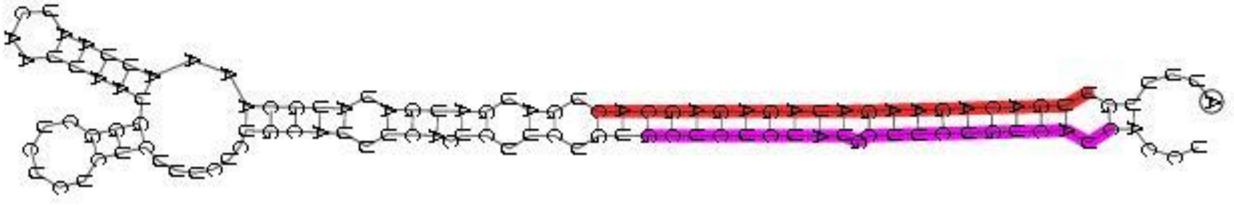

Jcu_MIR156a

```
 1 Jcu_miRNA156a      100.0%   TTCTGGGACACAGAAATTGACAGAAGAGAGTGAGCACACAGAGGCATTTGTATAAATCTATACCGTTGCTTTTGCGTGCTCACTTCTCTTTCTGTCAGTTTCCAGCGCCGGAA
 2 J25_233918_x1      100.0%   ---------------------------------------------------------------------------GTGCTCACTTCTCTTTCTGTCAGTT-------------
 3 J24_1945121_x3     100.0%   ---------------------------------------------------------------------------GTGCTCACTTCTCTTTCTGTCAGT--------------
 4 J24_191180_x53     100.0%   ----------------------------------------------------------------------------TGCTCACTTCTCTTTCTGTCAGTT-------------
 5 J23_483767_x3      100.0%   ---------------------------------------------------------------------------GTGCTCACTTCTCTTTCTGTCAG---------------
 6 J23_305455_x609    100.0%   ----------------------------------------------------------------------------TGCTCACTTCTCTTTCTGTCAGT--------------
 7 J23_278978_x2748   100.0%   -----------------------------------------------------------------------------GCTCACTTCTCTTTCTGTCAGTT-------------
 8 J24_526345_x17     100.0%   -----------------------------------------------------------------------------GCTCACTTCTCTTTCTGTCAGTTT------------
 9 J25_55967_x10      100.0%   -----------------------------------------------------------------------------GCTCACTTCTCTTTCTGTCAGTTTC-----------
10 J22_2023022_x1     100.0%   ------------------------------------------------------------------------------CTCACTTCTCTTTCTGTCAGTT-------------
11 J22_1461591_x129   100.0%   ---------------------------------------------------------------------------GTGCTCACTTCTCTTTCTGTCA----------------
12 J22_1460425_x130   100.0%   ----------------------------------------------------------------------------TGCTCACTTCTCTTTCTGTCAG---------------
13 J22_1229332_x5519  100.0%   -----------------------------------------------------------------------------GCTCACTTCTCTTTCTGTCAGT--------------
14 J21_2236809_x2     100.0%   ------------------------------------------------------------------------------CTCACTTCTCTTTCTGTCAGT--------------
15 J23_1057311_x1     100.0%   -------------------------------------------------------------------------GCGTGCTCACTTCTCTTTCTGTC-----------------
16 J22_1478001_x110   100.0%   --------------------------------------------------------------------------CGTGCTCACTTCTCTTTCTGTC-----------------
17 J21_1769670_x175   100.0%   -----------------------------------------------------------------------------GCTCACTTCTCTTTCTGTCAG---------------
18 J21_2686149_x1     100.0%   ---------------------------------------------------------------------------GTGCTCACTTCTCTTTCTGTC-----------------
19 J21_1994023_x19    100.0%   ----------------------------------------------------------------------------TGCTCACTTCTCTTTCTGTCA----------------
20 J23_1023175_x1     100.0%   -------------------------------------------------------------------------------TCACTTCTCTTTCTGTCAGTTTC-----------
21 J22_1962047_x1     100.0%   --------------------------------------------------------------------------------CACTTCTCTTTCTGTCAGTTTC-----------
22 J20_217013_x5      100.0%   -----------------------------------------------------------------------------GCTCACTTCTCTTTCTGTCA----------------
23 J20_213651_x7      100.0%   -------------------------------------------------------------------------------TCACTTCTCTTTCTGTCAGT--------------
24 J20_216196_x6      100.0%   --------------------------------------------------------------------------------CACTTCTCTTTCTGTCAGTT-------------
25 J20_260731_x1      100.0%   ----------------------------------------------------------------------------TGCTCACTTCTCTTTCTGTC-----------------
26 J19_180100_x6      100.0%   -----------------------------------------------------------------------------GCTCACTTCTCTTTCTGTC-----------------
27 J19_183667_x4      100.0%   --------------------------------------------------------------------------------CACTTCTCTTTCTGTCAGT--------------
28 J19_243595_x1      100.0%   ---------------------------------------------------------------------------------ACTTCTCTTTCTGTCAGTT-------------
29 J18_195954_x1      100.0%   ----------------------------------------------------------------------------------CTTCTCTTTCTGTCAGTT-------------
30 J18_163013_x1      100.0%   -----------------------------------------------------------------------------GCTCACTTCTCTTTCTGT------------------
31 J18_140755_x6      100.0%   ---------------------------------------------------------------------------------ACTTCTCTTTCTGTCAGT--------------
33 J23_355269_x30     100.0%   --------------AATTGACAGAAGAGAGTGAGCAC----------------------------------------------------------------------------
34 J22_1833704_x3     100.0%   ---------------ATTGACAGAAGAGAGTGAGCAC----------------------------------------------------------------------------
35 J21_997186_x16481  100.0%   ----------------TTGACAGAAGAGAGTGAGCAC----------------------------------------------------------------------------
36 J22_1807769_x4     100.0%   ----------------TTGACAGAAGAGAGTGAGCACA---------------------------------------------------------------------------
37 J20_158160_x254    100.0%   ----------------TTGACAGAAGAGAGTGAGCA-----------------------------------------------------------------------------
38 J20_75459_x11810   100.0%   -----------------TGACAGAAGAGAGTGAGCAC----------------------------------------------------------------------------
39 J21_1850539_x87    100.0%   -----------------TGACAGAAGAGAGTGAGCACA---------------------------------------------------------------------------
40 J19_162060_x57     100.0%   ----------------TTGACAGAAGAGAGTGAGC------------------------------------------------------------------------------
41 J20_215890_x6      100.0%   ------------------GACAGAAGAGAGTGAGCACA---------------------------------------------------------------------------
42 J19_129387_x729    100.0%   -----------------TGACAGAAGAGAGTGAGCA-----------------------------------------------------------------------------
43 J19_162866_x52     100.0%   ------------------GACAGAAGAGAGTGAGCAC----------------------------------------------------------------------------
44 J18_133614_x20     100.0%   ----------------TTGACAGAAGAGAGTGAG-------------------------------------------------------------------------------
45 J18_126662_x56     100.0%   -----------------TGACAGAAGAGAGTGAGC------------------------------------------------------------------------------
46 J19_159189_x76     100.0%   -------------------ACAGAAGAGAGTGAGCACA---------------------------------------------------------------------------
47 J20_192936_x35     100.0%   -------------------ACAGAAGAGAGTGAGCACAC--------------------------------------------------------------------------
48 J21_2275582_x1     100.0%   --------------------CAGAAGAGAGTGAGCACACAG------------------------------------------------------------------------
49 J18_149635_x2      100.0%   ------------------GACAGAAGAGAGTGAGCA-----------------------------------------------------------------------------
50 J18_142156_x5      100.0%   -------------------ACAGAAGAGAGTGAGCAC----------------------------------------------------------------------------
51 J18_160524_x1      100.0%   --------------------CAGAAGAGAGTGAGCACA---------------------------------------------------------------------------
```

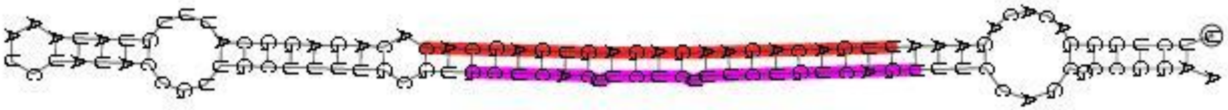

Jcu_MIR156b

```
 1 Jcu_miRNA156b     100.0%   ATTACTGAGAGAGACTGACAGAAGAGAGTGAGCACACGCAGGCAATTGTATGAGGCCATACCATTGTAGTGATGTGTGCTCACCTCTCTTCTGTCATCTT
 2 J22_1591836_x40   100.0%   ----------------------------------------------------------------------------TGCTCACCTCTCTTCTGTCATC--
 3 J22_1983613_x1    100.0%   -----------------------------------------------------------------------------GCTCACCTCTCTTCTGTCATCT-
 4 J19_217458_x1     100.0%   -----------------------------------------------------------------------------GCTCACCTCTCTTCTGTCA----
 5 J21_1956142_x30   100.0%   --------------CTGACAGAAGAGAGTGAGCAC-----------------------------------------------------------------
 6 J20_75459_x11810  100.0%   ---------------TGACAGAAGAGAGTGAGCAC-----------------------------------------------------------------
 7 J21_1850539_x87   100.0%   ---------------TGACAGAAGAGAGTGAGCACA----------------------------------------------------------------
 8 J20_215890_x6     100.0%   ----------------GACAGAAGAGAGTGAGCACA----------------------------------------------------------------
 9 J19_162866_x52    100.0%   ----------------GACAGAAGAGAGTGAGCAC-----------------------------------------------------------------
10 J19_129387_x729   100.0%   ---------------TGACAGAAGAGAGTGAGCA------------------------------------------------------------------
11 J18_126662_x56    100.0%   ---------------TGACAGAAGAGAGTGAGC-------------------------------------------------------------------
12 J19_159189_x76    100.0%   -----------------ACAGAAGAGAGTGAGCACA----------------------------------------------------------------
13 J20_192936_x35    100.0%   -----------------ACAGAAGAGAGTGAGCACAC---------------------------------------------------------------
14 J18_142156_x5     100.0%   -----------------ACAGAAGAGAGTGAGCAC-----------------------------------------------------------------
15 J18_149635_x2     100.0%   ----------------GACAGAAGAGAGTGAGCA------------------------------------------------------------------
16 J18_160524_x1     100.0%   ------------------CAGAAGAGAGTGAGCACA----------------------------------------------------------------
```

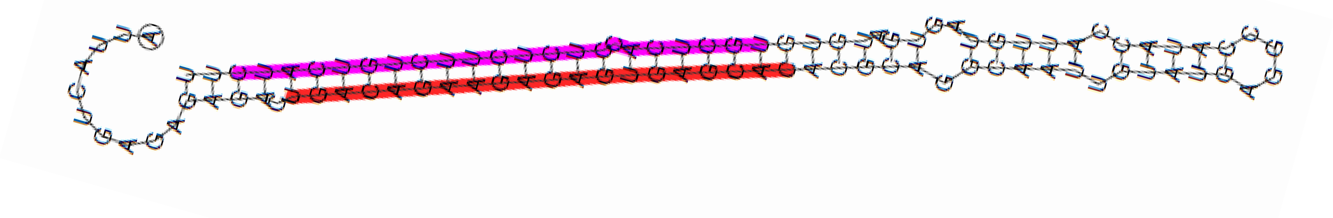

Jcu_MIR156c

```
 1 Jcu_miRNA156c     100.0%   GGAGGGTGACAGAAGAGAGTGAGCACACATGGTTTTCTTGCATTCATACTTCATGCTCGTAGCTCTGCGTGCTCACCCTCTATCTGTCATCCACCTCTCT
 2 J22_1951587_x1    100.0%   --------ACAGAAGAGAGTGAGCACACAT----------------------------------------------------------------------
 3 J21_2195375_x2    100.0%   ---------CAGAAGAGAGTGAGCACACAT----------------------------------------------------------------------
 4 J20_192936_x35    100.0%   --------ACAGAAGAGAGTGAGCACAC------------------------------------------------------------------------
 5 J20_224604_x3     100.0%   -----GTGACAGAAGAGAGTGAGCA---------------------------------------------------------------------------
 6 J21_1965158_x27   100.0%   -----GTGACAGAAGAGAGTGAGCAC--------------------------------------------------------------------------
 7 J20_75459_x11810  100.0%   ------TGACAGAAGAGAGTGAGCAC--------------------------------------------------------------------------
 8 J21_1850539_x87   100.0%   ------TGACAGAAGAGAGTGAGCACA-------------------------------------------------------------------------
 9 J20_215890_x6     100.0%   -------GACAGAAGAGAGTGAGCACA-------------------------------------------------------------------------
10 J19_162866_x52    100.0%   -------GACAGAAGAGAGTGAGCAC--------------------------------------------------------------------------
11 J19_159189_x76    100.0%   --------ACAGAAGAGAGTGAGCACA-------------------------------------------------------------------------
12 J18_149635_x2     100.0%   -------GACAGAAGAGAGTGAGCA---------------------------------------------------------------------------
13 J18_142156_x5     100.0%   --------ACAGAAGAGAGTGAGCAC--------------------------------------------------------------------------
14 J19_129387_x729   100.0%   ------TGACAGAAGAGAGTGAGCA---------------------------------------------------------------------------
15 J18_160524_x1     100.0%   ---------CAGAAGAGAGTGAGCACA-------------------------------------------------------------------------
16 J18_126662_x56    100.0%   ------TGACAGAAGAGAGTGAGC----------------------------------------------------------------------------
```

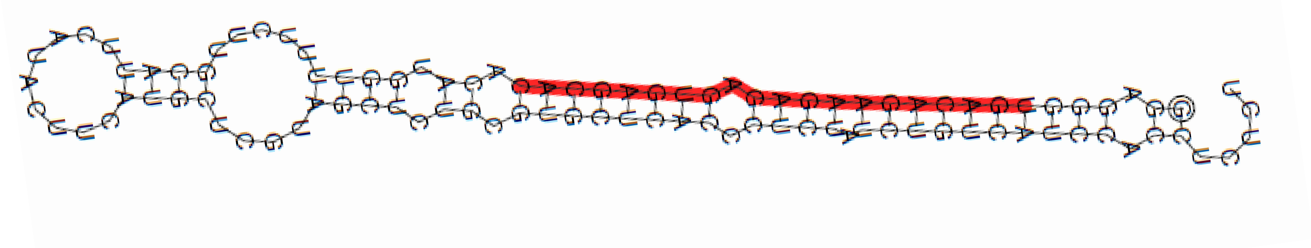

Jcu_MIR159

```
 1 Jcu_miRNA159      100.0%  AGGGTTAAGGAGTGGAGCTCCTTGAAGTCCAATAGAGGATCTTGCTAGGTTAGATTAAGCTGCTAAGCTATGCATCCCATAGCCCTATCCCATCTTGAAGAATATTTTTATTTGGATAGGCTTATGGCTTTCTTTTCTCAGGAGCTTTTTTACCTAACGTTAGATCCTTTTTTGGATTGAAGGGAGCTCTACACATCTCCCTCTTACA
 2 J25_193270_x1     100.0%  -----------GTGGAGCTCCTTGAAGTCCAATAGA----------------------------------------------------------------------------------------------------------------------------------------------------------------------------
 3 J22_1457279_x132  100.0%  --------------GAGCTCCTTGAAGTCCAATAGA----------------------------------------------------------------------------------------------------------------------------------------------------------------------------
 4 J21_2000167_x18   100.0%  --------------GAGCTCCTTGAAGTCCAATAG-----------------------------------------------------------------------------------------------------------------------------------------------------------------------------
 5 J21_1856235_x83   100.0%  ---------------AGCTCCTTGAAGTCCAATAGA----------------------------------------------------------------------------------------------------------------------------------------------------------------------------
 6 J20_184628_x60    100.0%  --------------GAGCTCCTTGAAGTCCAATA------------------------------------------------------------------------------------------------------------------------------------------------------------------------------
 7 J19_186230_x3     100.0%  --------------GAGCTCCTTGAAGTCCAAT-------------------------------------------------------------------------------------------------------------------------------------------------------------------------------
 8 J21_2424651_x1    100.0%  ------------------------------------------------------------------------------------------------------------------------------------------------------------------------TTTTTGGATTGAAGGGAGCTC-------------------
 9 J20_142107_x512   100.0%  --------------------------------------------------------------------------------------------------------------------------------------------------------------------------TTTGGATTGAAGGGAGCTCT------------------
10 J21_802764_x49102 100.0%  --------------------------------------------------------------------------------------------------------------------------------------------------------------------------TTTGGATTGAAGGGAGCTCTA-----------------
11 J22_1537092_x65   100.0%  --------------------------------------------------------------------------------------------------------------------------------------------------------------------------TTTGGATTGAAGGGAGCTCTAC----------------
12 J23_397817_x10    100.0%  --------------------------------------------------------------------------------------------------------------------------------------------------------------------------TTTGGATTGAAGGGAGCTCTACA---------------
13 J24_5586869_x1    100.0%  --------------------------------------------------------------------------------------------------------------------------------------------------------------------------TTTGGATTGAAGGGAGCTCTACAC--------------
14 J22_1831478_x3    100.0%  ---------------------------------------------------------------------------------------------------------------------------------------------------------------------------TTGGATTGAAGGGAGCTCTACA---------------
15 J21_2172021_x3    100.0%  ---------------------------------------------------------------------------------------------------------------------------------------------------------------------------TTGGATTGAAGGGAGCTCTAC----------------
16 J20_137410_x668   100.0%  ---------------------------------------------------------------------------------------------------------------------------------------------------------------------------TTGGATTGAAGGGAGCTCTA-----------------
17 J19_176573_x10    100.0%  ---------------------------------------------------------------------------------------------------------------------------------------------------------------------------TTGGATTGAAGGGAGCTCT------------------
18 J19_159856_x73    100.0%  --------------------------------------------------------------------------------------------------------------------------------------------------------------------------TTTGGATTGAAGGGAGCTC-------------------
19 J19_166699_x33    100.0%  ----------------------------------------------------------------------------------------------------------------------------------------------------------------------------TGGATTGAAGGGAGCTCTA-----------------
20 J18_138941_x8     100.0%  -----------------------------------------------------------------------------------------------------------------------------------------------------------------------------GGATTGAAGGGAGCTCTA-----------------
21 J18_108615_x426   100.0%  --------------------------------------------------------------------------------------------------------------------------------------------------------------------------TTTGGATTGAAGGGAGCT--------------------
```

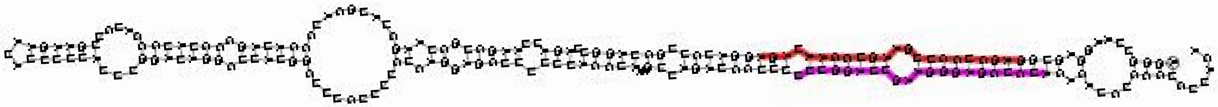

Jcu_MIR1463

```
1 Jcu_miR1463     100.0%   AAAAGTTCTGGATGTCCTTGTTTGGGTGTTTATTGTTACTTGTTATATCAAATCTTCTATGATGGATTTCTGAGAACTTTATT
2 J25_57780_x8    100.0%   -----------------------------------------------------CTTCTATGATGGATTTCTGAGAACT-----
3 J24_5360430_x1  100.0%   -----------------------------------------------------CTTCTATGATGGATTTCTGAGAAC------
4 J24_1865063_x3  100.0%   ------------------------------------------------------TTCTATGATGGATTTCTGAGAACT-----
5 J23_619240_x2   100.0%   ------------------------------------------------------TTCTATGATGGATTTCTGAGAAC------
```

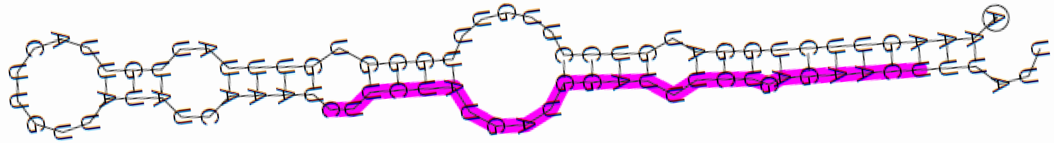

Jcu_MIR166a

```
 1 Jcu_miRNA166a      100.0%  TTTCTCTTTTGAGGGGAATGTTGTCTGGCTCGAGGACTCTCTTTTGATCCATCTTTGTTGATGATTTTTCTTTAATTATATCCATAGATCTAATAGGTGTATGGATTTAGAATTAGATTGATCATGTGATAGTGTCGTCGGACCAGGCTTCATTCCCC--
 2 J24_2094014_x3     100.0%  -------TTTGAGGGGAATGTTGTCTGGCTC---------------------------------------------------------------------------------------------------------------------------------
 3 J21_2038316_x12    100.0%  -------TTTGAGGGGAATGTTGTCTGG------------------------------------------------------------------------------------------------------------------------------------
 4 J21_2283867_x1     100.0%  --------TTGAGGGGAATGTTGTCTGGC-----------------------------------------------------------------------------------------------------------------------------------
 5 J21_2574016_x1     100.0%  ----------GAGGGGAATGTTGTCTGGCTC---------------------------------------------------------------------------------------------------------------------------------
 6 J20_253565_x1      100.0%  ------------GGGGAATGTTGTCTGGCTCG--------------------------------------------------------------------------------------------------------------------------------
 7 J21_2308024_x1     100.0%  ------------GGGGAATGTTGTCTGGCTCGA-------------------------------------------------------------------------------------------------------------------------------
 8 J19_182163_x4      100.0%  --------------GGAATGTTGTCTGGCTCGA-------------------------------------------------------------------------------------------------------------------------------
 9 J20_210506_x9      100.0%  --------------GGAATGTTGTCTGGCTCGAG------------------------------------------------------------------------------------------------------------------------------
10 J21_1384537_x2286  100.0%  --------------GGAATGTTGTCTGGCTCGAGG-----------------------------------------------------------------------------------------------------------------------------
11 J22_1660145_x21    100.0%  --------------GGAATGTTGTCTGGCTCGAGGA----------------------------------------------------------------------------------------------------------------------------
12 J23_1151125_x1     100.0%  --------------GGAATGTTGTCTGGCTCGAGGAC---------------------------------------------------------------------------------------------------------------------------
13 J24_2099921_x3     100.0%  --------------GGAATGTTGTCTGGCTCGAGGACT--------------------------------------------------------------------------------------------------------------------------
14 J22_1952194_x1     100.0%  ---------------GAATGTTGTCTGGCTCGAGGAC---------------------------------------------------------------------------------------------------------------------------
15 J21_1994650_x19    100.0%  ---------------GAATGTTGTCTGGCTCGAGGA----------------------------------------------------------------------------------------------------------------------------
16 J20_214516_x6      100.0%  ---------------GAATGTTGTCTGGCTCGAGG-----------------------------------------------------------------------------------------------------------------------------
17 J21_1851315_x86    100.0%  ----------------------------------------------------------------------------------------------------------------------------------------GTCGGACCAGGCTTCATTCCC---
18 J22_1804949_x4     100.0%  ----------------------------------------------------------------------------------------------------------------------------------------GTCGGACCAGGCTTCATTCCCC--
19 J21_554760_x105664 100.0%  -----------------------------------------------------------------------------------------------------------------------------------------TCGGACCAGGCTTCATTCCCC--
20 J22_1531901_x68     95.5%  -----------------------------------------------------------------------------------------------------------------------------------------TCGGACCAGGCTTCATTCCCCC-
21 J23_756083_x1       91.3%  -----------------------------------------------------------------------------------------------------------------------------------------TCGGACCAGGCTTCATTCCCCCC
22 J21_2370376_x1      95.2%  ------------------------------------------------------------------------------------------------------------------------------------------CGGACCAGGCTTCATTCCCCC-
23 J20_105367_x3808   100.0%  -----------------------------------------------------------------------------------------------------------------------------------------TCGGACCAGGCTTCATTCCC---
24 J20_153381_x305    100.0%  ------------------------------------------------------------------------------------------------------------------------------------------CGGACCAGGCTTCATTCCCC--
25 J19_155651_x114    100.0%  -------------------------------------------------------------------------------------------------------------------------------------------GGACCAGGCTTCATTCCCC--
26 J19_173882_x14     100.0%  ------------------------------------------------------------------------------------------------------------------------------------------CGGACCAGGCTTCATTCCC---
27 J19_151294_x161    100.0%  -----------------------------------------------------------------------------------------------------------------------------------------TCGGACCAGGCTTCATTCC----
28 J18_131802_x29     100.0%  -----------------------------------------------------------------------------------------------------------------------------------------TCGGACCAGGCTTCATTC-----
29 J18_141626_x5      100.0%  -------------------------------------------------------------------------------------------------------------------------------------------GGACCAGGCTTCATTCCC---
30 J18_218824_x1      100.0%  ------------------------------------------------------------------------------------------------------------------------------------------CGGACCAGGCTTCATTCC----
31 J18_150685_x2      100.0%  --------------------------------------------------------------------------------------------------------------------------------------------GACCAGGCTTCATTCCCC--
```

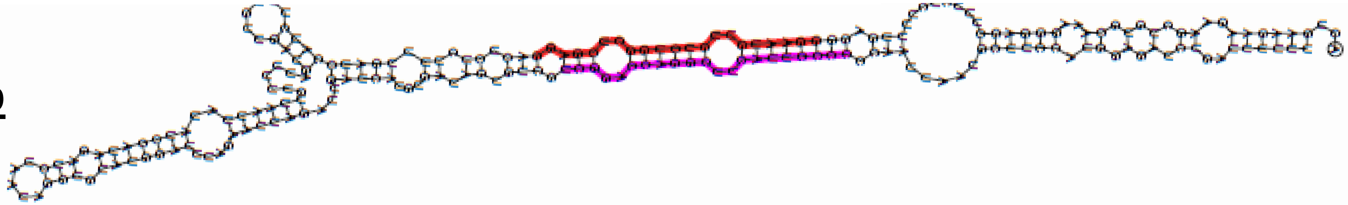

Jcu_MIR166b

```
 1 Jcu_miR166b        100.0%  AGAAGCTGTATATCTTTTGAGGGGAATGTTGTCTGGCTCGAGGCCACTAACTAGATCTACAAGTTTATCTCAATTGATTTTCTTTTTCA
 2 J24_2094014_x3     100.0%  ---------------TTTGAGGGGAATGTTGTCTGGCTC--------------------------------------------------
 3 J21_2038316_x12    100.0%  ---------------TTTGAGGGGAATGTTGTCTGG-----------------------------------------------------
 4 J21_2283867_x1     100.0%  ----------------TTGAGGGGAATGTTGTCTGGC----------------------------------------------------
 5 J21_2574016_x1     100.0%  ------------------GAGGGGAATGTTGTCTGGCTC--------------------------------------------------
 6 J20_253565_x1      100.0%  --------------------GGGGAATGTTGTCTGGCTCG-------------------------------------------------
 7 J21_2308024_x1     100.0%  --------------------GGGGAATGTTGTCTGGCTCGA------------------------------------------------
 8 J19_182163_x4      100.0%  ----------------------GGAATGTTGTCTGGCTCGA------------------------------------------------
 9 J20_210506_x9      100.0%  ----------------------GGAATGTTGTCTGGCTCGAG-----------------------------------------------
10 J21_1384537_x2286  100.0%  ----------------------GGAATGTTGTCTGGCTCGAGG----------------------------------------------
11 J22_1785069_x5     100.0%  ----------------------GGAATGTTGTCTGGCTCGAGGC---------------------------------------------
12 J21_2171646_x3     100.0%  -----------------------GAATGTTGTCTGGCTCGAGGC---------------------------------------------
13 J22_1911659_x1     100.0%  -----------------------GAATGTTGTCTGGCTCGAGGCC--------------------------------------------
14 J20_214516_x6      100.0%  -----------------------GAATGTTGTCTGGCTCGAGG----------------------------------------------
15 J21_2648432_x1     100.0%  -------------------------ATGTTGTCTGGCTCGAGGCCA-------------------------------------------
```

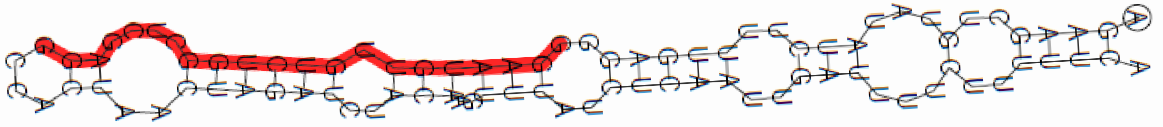

Jcu_MIR167a

```
 1 Jcu_miRNA167a     100.0%  ACCTCTAGTAGTTGAAGCTGCCAGCATGATCTGAGCTTTCCTTAATACTCATCGATCCTACCCTATCCAGAGAGGAGGGATTAGATCATGTGGCAGTTTCACCTGTTAATGGTA
 2 J25_261788_x1     100.0%  ------------TGAAGCTGCCAGCATGATCTGAGCT-----------------------------------------------------------------------------
 3 J24_4921698_x1    100.0%  ------------TGAAGCTGCCAGCATGATCTGAGC------------------------------------------------------------------------------
 4 J23_427162_x6     100.0%  -----------TTGAAGCTGCCAGCATGATCTGA--------------------------------------------------------------------------------
 5 J23_326151_x128   100.0%  ------------TGAAGCTGCCAGCATGATCTGAG-------------------------------------------------------------------------------
 6 J22_1809197_x4    100.0%  -----------TTGAAGCTGCCAGCATGATCTG---------------------------------------------------------------------------------
 7 J22_1863832_x2    100.0%  --------------AAGCTGCCAGCATGATCTGAGC------------------------------------------------------------------------------
 8 J22_0_x829649     100.0%  ------------TGAAGCTGCCAGCATGATCTGA--------------------------------------------------------------------------------
 9 J22_1797004_x5    100.0%  ----------GTTGAAGCTGCCAGCATGATCT----------------------------------------------------------------------------------
10 J21_1484832_x1335 100.0%  -------------GAAGCTGCCAGCATGATCTGA--------------------------------------------------------------------------------
11 J21_961495_x18100 100.0%  ------------TGAAGCTGCCAGCATGATCTG---------------------------------------------------------------------------------
12 J21_2263849_x2    100.0%  ----------GTTGAAGCTGCCAGCATGATC-----------------------------------------------------------------------------------
13 J20_192364_x36    100.0%  --------------AAGCTGCCAGCATGATCTGA--------------------------------------------------------------------------------
14 J20_171675_x127   100.0%  ------------TGAAGCTGCCAGCATGATCT----------------------------------------------------------------------------------
15 J20_219243_x5     100.0%  -------------GAAGCTGCCAGCATGATCTG---------------------------------------------------------------------------------
16 J19_151129_x165   100.0%  ---------------AGCTGCCAGCATGATCTGA--------------------------------------------------------------------------------
17 J19_163275_x50    100.0%  ------------TGAAGCTGCCAGCATGATC-----------------------------------------------------------------------------------
18 J18_117586_x196   100.0%  ------------TGAAGCTGCCAGCATGAT------------------------------------------------------------------------------------
19 J18_121420_x134   100.0%  ----------------GCTGCCAGCATGATCTGA--------------------------------------------------------------------------------
20 J21_2052311_x10   100.0%  --------------------------------------------------------------------------------TTAGATCATGTGGCAGTTTCA-------------
21 J24_4106312_x1    100.0%  --------------------------------------------------------------------------------TTAGATCATGTGGCAGTTTCACCT----------
22 J22_1859016_x2    100.0%  ----------------------------------------------------------------------------------AGATCATGTGGCAGTTTCACCT----------
23 J21_1821946_x110  100.0%  ----------------------------------------------------------------------------------AGATCATGTGGCAGTTTCACC-----------
24 J21_2147751_x3    100.0%  -----------------------------------------------------------------------------------GATCATGTGGCAGTTTCACCT----------
25 J20_223905_x3     100.0%  ------------------------------------------------------------------------------------ATCATGTGGCAGTTTCACCT----------
26 J20_193415_x33    100.0%  -----------------------------------------------------------------------------------GATCATGTGGCAGTTTCACC-----------
27 J20_229476_x2     100.0%  ----------------------------------------------------------------------------------AGATCATGTGGCAGTTTCAC------------
28 J21_2094654_x6    100.0%  ------------------------------------------------------------------------------------ATCATGTGGCAGTTTCACCTG---------
29 J19_190904_x2     100.0%  -------------------------------------------------------------------------------------TCATGTGGCAGTTTCACCT----------
```

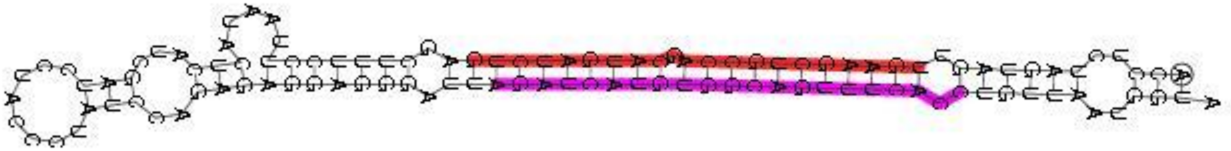

Jcu_MIR167b

```
 1 Jcu_miRNA167b      100.0%  GTTGAAGCTGCCAGCATGATCTAATCTTCCTTTCTCCTCCTCCGTCGTCGTCGGAAGGCAAGATCAGATCATGTGGTAGCTTCACCTGTTGATGGCTTCACGGCAA
 2 J24_4395384_x1     100.0%  ---------------------------------------------------------------TCAGATCATGTGGTAGCTTCACCT-------------------
 3 J21_1723896_x252   100.0%  ---------------------------------------------------------------TCAGATCATGTGGTAGCTTCA----------------------
 4 J20_226062_x3      100.0%  ---------------------------------------------------------------TCAGATCATGTGGTAGCTTC-----------------------
 5 J21_2522965_x1     100.0%  -----------------------------------------------------------------AGATCATGTGGTAGCTTCACC--------------------
 6 J21_2564152_x1     100.0%  -----------------------------------------------------------AAGATCAGATCATGTGGTAGC--------------------------
 7 J21_2099934_x6     100.0%  ------------------------------------------------------------AGATCAGATCATGTGGTAGCT-------------------------
 8 J20_281001_x1      100.0%  -------------------------------------------------------------GATCAGATCATGTGGTAGCT-------------------------
 9 J22_1837700_x3     100.0%  ----------------------------------------------------------------------ATGTGGTAGCTTCACCTGTTGA--------------
10 J21_2222627_x2     100.0%  -----------------------------------------------------------------------TGTGGTAGCTTCACCTGTTGA--------------
11 J21_2263849_x2     100.0%  GTTGAAGCTGCCAGCATGATC-------------------------------------------------------------------------------------
12 J22_1797004_x5     100.0%  GTTGAAGCTGCCAGCATGATCT------------------------------------------------------------------------------------
13 J21_1271883_x3706 100.0%  --TGAAGCTGCCAGCATGATCTA-----------------------------------------------------------------------------------
14 J22_1246151_x2517 100.0%  --TGAAGCTGCCAGCATGATCTAA----------------------------------------------------------------------------------
15 J23_353628_x32    100.0%  --TGAAGCTGCCAGCATGATCTAAT---------------------------------------------------------------------------------
16 J24_3421511_x1    100.0%  --TGAAGCTGCCAGCATGATCTAATC--------------------------------------------------------------------------------
17 J22_1878184_x2    100.0%  ---GAAGCTGCCAGCATGATCTAAT---------------------------------------------------------------------------------
18 J21_2056529_x9    100.0%  ---GAAGCTGCCAGCATGATCTAA----------------------------------------------------------------------------------
19 J22_2090397_x1    100.0%  ----AAGCTGCCAGCATGATCTAATC--------------------------------------------------------------------------------
20 J20_171675_x127   100.0%  --TGAAGCTGCCAGCATGATCT------------------------------------------------------------------------------------
21 J19_257411_x1     100.0%  -----AGCTGCCAGCATGATCTAA----------------------------------------------------------------------------------
22 J19_163275_x50    100.0%  --TGAAGCTGCCAGCATGATC-------------------------------------------------------------------------------------
23 J18_117586_x196   100.0%  --TGAAGCTGCCAGCATGAT--------------------------------------------------------------------------------------
24 J18_143816_x3     100.0%  -----AGCTGCCAGCATGATCTA-----------------------------------------------------------------------------------
```

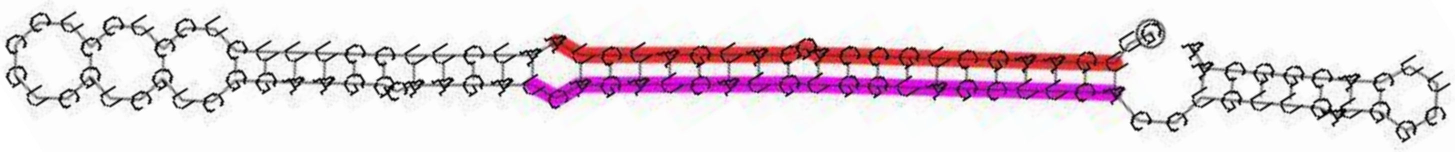

Jcu_MIR168

```
 1 Jcu_miRNA168       100.0%  AGTTGATTCTGTTACCGGCGGTCTCTAATTCGCTTGGTGCAGGTCGGGAACTGATTGGCTTCGCTTGTATGTACTTGTGTGTACAGTATGTACTTAATCAATGCCAGATGGCGAGATGTGATTAGTTGAGATGAATGGAAGCGAATTGGATCCCGCCTTGCATCAACTGAATCGGAGACCGCGGTGAAGGGT
 2 J24_2518093_x2     100.0%  ----------------------------------------------------------------------------------------------------------------------------------------------------GATCCCGCCTTGCATCAACTGAAT--------------------
 3 J21_2273095_x1     100.0%  ------------------------------------------------------------------------------------------------------------------------------------------------------TCCCGCCTTGCATCAACTGAA---------------------
 4 J21_1881254_x64    100.0%  -------------------------------------------------------------------------------------------------------------------------------------------------------CCCGCCTTGCATCAACTGAAT--------------------
 5 J20_198378_x23     100.0%  -------------------------------------------------------------------------------------------------------------------------------------------------------CCCGCCTTGCATCAACTGAA---------------------
 6 J20_252538_x1      100.0%  --------------------------------------------------------------------------------------------------------------------------------------------------------CCGCCTTGCATCAACTGAAT--------------------
 7 J22_1772125_x6     100.0%  ---------------------------------------------------------------------------------------------------------------------------------------------------------CGCCTTGCATCAACTGAATCGG-----------------
 8 J21_2236375_x2     100.0%  ----------------------------------------------------------------------------------------------------------------------------------------------------------GCCTTGCATCAACTGAATCGG-----------------
 9 J19_177774_x8      100.0%  -------------------------------------------------------------------------------------------------------------------------------------------------------CCCGCCTTGCATCAACTGA----------------------
10 J21_2128831_x4     100.0%  ------------------------------------------------------------------------------------------------------------------------------------------------------------CTTGCATCAACTGAATCGGAG---------------
11 J22_1608235_x35    100.0%  -----------------------------------------------------------------------------------------------------------------------------------------------------------CCTTGCATCAACTGAATCGGAG---------------
12 J23_601964_x2      100.0%  -----------------------------------------------------------------------------------------------------------------------------------------------------------CCTTGCATCAACTGAATCGGAGA--------------
13 J24_4047663_x1     100.0%  -----------------------------------------------------------------------------------------------------------------------------------------------------------CCTTGCATCAACTGAATCGGAGAC-------------
14 J21_2153736_x3     100.0%  -----------------------------------------------------------------------------------------------------------------------------------------------------------CCTTGCATCAACTGAATCGGA----------------
15 J21_1742316_x217   100.0%  -------------------------------------------------------------------------------------------------------------------------------------------------------------TTGCATCAACTGAATCGGAGA--------------
16 J22_1758462_x7     100.0%  -------------------------------------------------------------------------------------------------------------------------------------------------------------TTGCATCAACTGAATCGGAGAC-------------
17 J20_297874_x1      100.0%  -------------------------------------------------------------------------------------------------------------------------------------------------------------TTGCATCAACTGAATCGGAG---------------
18 J21_2075143_x8     100.0%  --------------------------------------------------------------------------------------------------------------------------------------------------------------TGCATCAACTGAATCGGAGAC-------------
19 J20_306027_x1      100.0%  --------------------------------------------------------------------------------------------------------------------------------------------------------------TGCATCAACTGAATCGGAGA--------------
20 J18_194121_x1      100.0%  -------------------------------------------------------------------------------------------------------------------------------------------------------------TTGCATCAACTGAATCGG-----------------
21 J19_187458_x2      100.0%  -------------------------------------------------------------------------------------------------------------------------------------------------------------TTGCATCAACTGAATCGGA----------------
22 J20_232000_x2      100.0%  ---------------------------------------------------------------------------------------------------------------------------------------------------------------GCATCAACTGAATCGGAGAC-------------
23 J21_1993368_x20    100.0%  ---------------------------------------------------------------------------------------------------------------------------------------------------------------GCATCAACTGAATCGGAGACC------------
24 J20_237598_x2      100.0%  ----------------------------------------------------------------------------------------------------------------------------------------------------------------CATCAACTGAATCGGAGACC------------
28 J18_144323_x3      100.0%  ---------------------TCTCTAATTCGCTTGGTG---------------------------------------------------------------------------------------------------------------------------------------------------------
29 J19_286613_x1      100.0%  ---------------------TCTCTAATTCGCTTGGTGC--------------------------------------------------------------------------------------------------------------------------------------------------------
30 J20_217608_x5      100.0%  ---------------------TCTCTAATTCGCTTGGTGCA-------------------------------------------------------------------------------------------------------------------------------------------------------
31 J21_1626098_x570   100.0%  ---------------------TCTCTAATTCGCTTGGTGCAG------------------------------------------------------------------------------------------------------------------------------------------------------
32 J22_1696554_x15    100.0%  ---------------------TCTCTAATTCGCTTGGTGCAGG-----------------------------------------------------------------------------------------------------------------------------------------------------
33 J23_943224_x1      100.0%  ---------------------TCTCTAATTCGCTTGGTGCAGGT----------------------------------------------------------------------------------------------------------------------------------------------------
34 J22_1815633_x4     100.0%  ----------------------CTCTAATTCGCTTGGTGCAGGT----------------------------------------------------------------------------------------------------------------------------------------------------
35 J21_2018960_x15    100.0%  ----------------------CTCTAATTCGCTTGGTGCAGG-----------------------------------------------------------------------------------------------------------------------------------------------------
36 J21_2123431_x4     100.0%  -----------------------TCTAATTCGCTTGGTGCAGGT----------------------------------------------------------------------------------------------------------------------------------------------------
37 J20_303861_x1      100.0%  ----------------------CTCTAATTCGCTTGGTGCAG------------------------------------------------------------------------------------------------------------------------------------------------------
38 J20_239870_x2      100.0%  -----------------------TCTAATTCGCTTGGTGCAGG-----------------------------------------------------------------------------------------------------------------------------------------------------
39 J19_185138_x3      100.0%  -----------------------TCTAATTCGCTTGGTGCAG------------------------------------------------------------------------------------------------------------------------------------------------------
40 J18_226664_x1      100.0%  -----------------------TCTAATTCGCTTGGTGCA-------------------------------------------------------------------------------------------------------------------------------------------------------
41 J21_1948874_x32    100.0%  --------------------------AATTCGCTTGGTGCAGGTCGG-------------------------------------------------------------------------------------------------------------------------------------------------
42 J22_1724468_x11    100.0%  --------------------------AATTCGCTTGGTGCAGGTCGGG------------------------------------------------------------------------------------------------------------------------------------------------
43 J24_1678432_x4     100.0%  --------------------------AATTCGCTTGGTGCAGGTCGGGAA----------------------------------------------------------------------------------------------------------------------------------------------
44 J21_2691109_x1     100.0%  ---------------------------ATTCGCTTGGTGCAGGTCGGG------------------------------------------------------------------------------------------------------------------------------------------------
45 J21_2685255_x1     100.0%  ----------------------------TTCGCTTGGTGCAGGTCGGGA-----------------------------------------------------------------------------------------------------------------------------------------------
46 J20_156306_x272    100.0%  -----------------------------TCGCTTGGTGCAGGTCGGGA-----------------------------------------------------------------------------------------------------------------------------------------------
47 J21_941900_x19595  100.0%  -----------------------------TCGCTTGGTGCAGGTCGGGAA----------------------------------------------------------------------------------------------------------------------------------------------
48 J22_1536247_x65    100.0%  -----------------------------TCGCTTGGTGCAGGTCGGGAAC---------------------------------------------------------------------------------------------------------------------------------------------
49 J24_1715272_x4     100.0%  -----------------------------TCGCTTGGTGCAGGTCGGGAACTG-------------------------------------------------------------------------------------------------------------------------------------------
50 J21_1962816_x28    100.0%  ------------------------------CGCTTGGTGCAGGTCGGGAAC---------------------------------------------------------------------------------------------------------------------------------------------
51 J20_194726_x31     100.0%  ------------------------------CGCTTGGTGCAGGTCGGGAA----------------------------------------------------------------------------------------------------------------------------------------------
52 J19_175640_x11     100.0%  -----------------------------TCGCTTGGTGCAGGTCGGG------------------------------------------------------------------------------------------------------------------------------------------------
53 J18_195618_x1      100.0%  -------------------------------GCTTGGTGCAGGTCGGGA-----------------------------------------------------------------------------------------------------------------------------------------------
54 J19_170889_x21     100.0%  -------------------------------GCTTGGTGCAGGTCGGGAA----------------------------------------------------------------------------------------------------------------------------------------------
55 J18_144971_x3      100.0%  -----------------------------TCGCTTGGTGCAGGTCGG-------------------------------------------------------------------------------------------------------------------------------------------------
56 J18_142785_x4      100.0%  --------------------------------CTTGGTGCAGGTCGGGAA----------------------------------------------------------------------------------------------------------------------------------------------
57 J21_2356363_x1     100.0%  ----------------------------------------AGGTCGGGAACTGATTGGCTT-----------------------------------------------------------------------------------------------------------------------------------
58 J21_2386272_x1     100.0%  -----------------------------------------GGTCGGGAACTGATTGGCTTC----------------------------------------------------------------------------------------------------------------------------------
59 J21_2608619_x1     100.0%  --------------------------------------------CGGGAACTGATTGGCTTCGCT-------------------------------------------------------------------------------------------------------------------------------
60 J22_1873372_x2     100.0%  --------------------------------------------CGGGAACTGATTGGCTTCGCTT------------------------------------------------------------------------------------------------------------------------------
61 J21_2119296_x5     100.0%  --------------------------------CTTGGTGCAGGTCGGGAACTG-------------------------------------------------------------------------------------------------------------------------------------------
62 J21_2292636_x1     100.0%  ---------------------------------TTGGTGCAGGTCGGGAACTGA------------------------------------------------------------------------------------------------------------------------------------------
63 J21_2003559_x17    100.0%  ----------------------------------TGGTGCAGGTCGGGAACTGAT-----------------------------------------------------------------------------------------------------------------------------------------
64 J22_2084690_x1     100.0%  ----------------------------------TGGTGCAGGTCGGGAACTGATT----------------------------------------------------------------------------------------------------------------------------------------
65 J24_2958651_x2     100.0%  ----------------------------------TGGTGCAGGTCGGGAACTGATTGG--------------------------------------------------------------------------------------------------------------------------------------
66 J23_587190_x2      100.0%  -----------------------------------GGTGCAGGTCGGGAACTGATTGG--------------------------------------------------------------------------------------------------------------------------------------
67 J20_248751_x1      100.0%  -----------------------------------GGTGCAGGTCGGGAACTGAT-----------------------------------------------------------------------------------------------------------------------------------------
68 J20_215050_x6      100.0%  ----------------------------------TGGTGCAGGTCGGGAACTGA------------------------------------------------------------------------------------------------------------------------------------------
69 J18_178071_x1      100.0%  -------------------------------------TGCAGGTCGGGAACTGAT-----------------------------------------------------------------------------------------------------------------------------------------
```

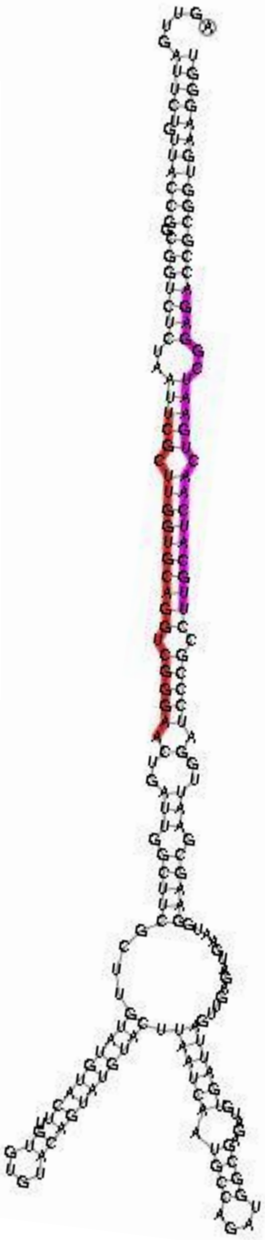

Jcu_MIR169a

```
 1 Jcu_miRNA169       100.0%  GCCTTGCATGAAGAGGTAGAGAGTGTAATTCAGCCAAGGATGACTTGCCGGCCTGGCCTTGCCTGCAGCTATCTACAACACCCGCCAACGCCGGCAAGTTGTTTCTTTGGCTACATTGTACTCTTTTCTTCTCATGTCAGG
 2 J23_392903_x11     100.0%  -------------------------------------------------------------------------------------------CGGCAAGTTGTTTCTTTGGCTAC---------------------------
 3 J23_425358_x7      100.0%  --------------------------------------------------------------------------------------------GGCAAGTTGTTTCTTTGGCTACA--------------------------
 4 J22_2125299_x1     100.0%  -------------------------------------------------------------------------------------------CGGCAAGTTGTTTCTTTGGCTA----------------------------
 5 J22_1660964_x21    100.0%  ---------------------------------------------------------------------------------------------GCAAGTTGTTTCTTTGGCTACA--------------------------
 6 J22_1643705_x25    100.0%  --------------------------------------------------------------------------------------------GGCAAGTTGTTTCTTTGGCTAC---------------------------
 7 J21_1989988_x20    100.0%  ---------------------------------------------------------------------------------------------GCAAGTTGTTTCTTTGGCTAC---------------------------
 8 J21_1947818_x32    100.0%  --------------------------------------------------------------------------------------------GGCAAGTTGTTTCTTTGGCTA----------------------------
 9 J20_216214_x6      100.0%  ---------------------------------------------------------------------------------------------GCAAGTTGTTTCTTTGGCTA----------------------------
10 J20_212314_x8      100.0%  --------------------------------------------------------------------------------------------GGCAAGTTGTTTCTTTGGCT-----------------------------
11 J19_273146_x1      100.0%  ---------------------------------------------------------------------------------------------GCAAGTTGTTTCTTTGGCT-----------------------------
12 J19_197298_x1      100.0%  --------------------------------------------------------------------------------------------GGCAAGTTGTTTCTTTGGC------------------------------
13 J18_206927_x1      100.0%  ---------------------------------------------------------------------------------------------GCAAGTTGTTTCTTTGGC------------------------------
15 J18_148293_x2      100.0%  ------------------------------CAGCCAAGGATGACTTGC---------------------------------------------------------------------------------------------
16 J22_2110946_x1     100.0%  -----------------------------TCAGCCAAGGATGACTTGCCGG------------------------------------------------------------------------------------------
17 J21_1251650_x4275  100.0%  ------------------------------CAGCCAAGGATGACTTGCCGG------------------------------------------------------------------------------------------
18 J22_1880218_x2     100.0%  ------------------------------CAGCCAAGGATGACTTGCCGGC-----------------------------------------------------------------------------------------
19 J20_145417_x443    100.0%  -------------------------------AGCCAAGGATGACTTGCCGG------------------------------------------------------------------------------------------
20 J20_241978_x2      100.0%  ------------------------------CAGCCAAGGATGACTTGCCG-------------------------------------------------------------------------------------------
21 J21_1953742_x30    100.0%  -------------------------------AGCCAAGGATGACTTGCCGGC-----------------------------------------------------------------------------------------
22 J19_191222_x2      100.0%  --------------------------------GCCAAGGATGACTTGCCGG------------------------------------------------------------------------------------------
```

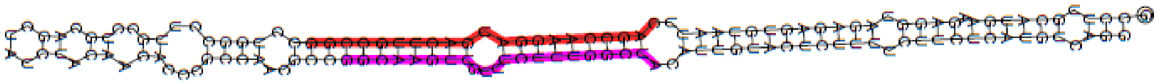

Jcu_MIR390

```
 1 Jcu_miR390        100.0%  GTATGGAAGAATCTGTTAAGCTCAGGAGGGATAGCGCCATAATCGATCATTACTCTTTTTTTCCTTGGGGTTTTGATTTTCTTTCTATAGCGCTATCCATCCTGAGTTTCATGGCTTCTTCTTGCTG
 2 J22_2076988_x1    100.0%  -----------------AAGCTCAGGAGGGATAGCGCCA----------------------------------------------------------------------------------------
 3 J21_1635946_x528  100.0%  -----------------AAGCTCAGGAGGGATAGCGCC-----------------------------------------------------------------------------------------
 4 J21_2651976_x1    100.0%  ------------------AGCTCAGGAGGGATAGCGCCA----------------------------------------------------------------------------------------
 5 J20_200267_x20    100.0%  -----------------AAGCTCAGGAGGGATAGCGC------------------------------------------------------------------------------------------
 6 J20_183883_x63    100.0%  ------------------AGCTCAGGAGGGATAGCGCC-----------------------------------------------------------------------------------------
 7 J19_224379_x1     100.0%  -----------------AAGCTCAGGAGGGATAGCG-------------------------------------------------------------------------------------------
 8 J19_250125_x1     100.0%  ------------------AGCTCAGGAGGGATAGCGC------------------------------------------------------------------------------------------
 9 J19_185798_x3     100.0%  -------------------GCTCAGGAGGGATAGCGCC-----------------------------------------------------------------------------------------
10 J18_214754_x1     100.0%  -----------------AAGCTCAGGAGGGATAGC--------------------------------------------------------------------------------------------
11 J18_223911_x1     100.0%  --------------------CTCAGGAGGGATAGCGCC-----------------------------------------------------------------------------------------
12 J18_144518_x3     100.0%  ------------------------------------------------------------------------------------------CGCTATCCATCCTGAGTT-------------------
13 J19_268572_x1     100.0%  ------------------------------------------------------------------------------------------CGCTATCCATCCTGAGTTT------------------
14 J20_185741_x56    100.0%  ------------------------------------------------------------------------------------------CGCTATCCATCCTGAGTTTC-----------------
15 J21_1844115_x92   100.0%  ------------------------------------------------------------------------------------------CGCTATCCATCCTGAGTTTCA----------------
16 J19_275800_x1     100.0%  -------------------------------------------------------------------------------------------GCTATCCATCCTGAGTTTC-----------------
```

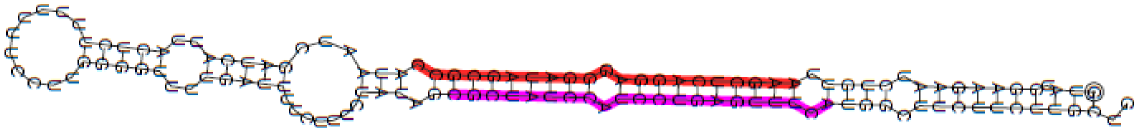

Jcu_MIR320

```
1 Jcu_miR320        100.0%  TTAAAATTACCGAACCGGGACGTGAAGGCTAACGGCGACGTTAGGGGGTCCGGGGACGGCGGCGGGGGACTCGGGAAGAGGTATCTTTTCTTTTTGATAG
2 J21_1930029_x39   100.0%  ----------CCGAACCGGGACGTGAAGGCT---------------------------------------------------------------------
3 J20_198286_x23    100.0%  ----------CCGAACCGGGACGTGAAGGC----------------------------------------------------------------------
4 J20_194937_x30    100.0%  -----------CGAACCGGGACGTGAAGGCT---------------------------------------------------------------------
5 J19_180172_x6     100.0%  ----------CCGAACCGGGACGTGAAGG-----------------------------------------------------------------------
6 J19_179290_x6     100.0%  -----------CGAACCGGGACGTGAAGGC----------------------------------------------------------------------
7 J18_144941_x3     100.0%  ----------CCGAACCGGGACGTGAAG------------------------------------------------------------------------
8 J18_144887_x3     100.0%  -----------CGAACCGGGACGTGAAGG-----------------------------------------------------------------------
```

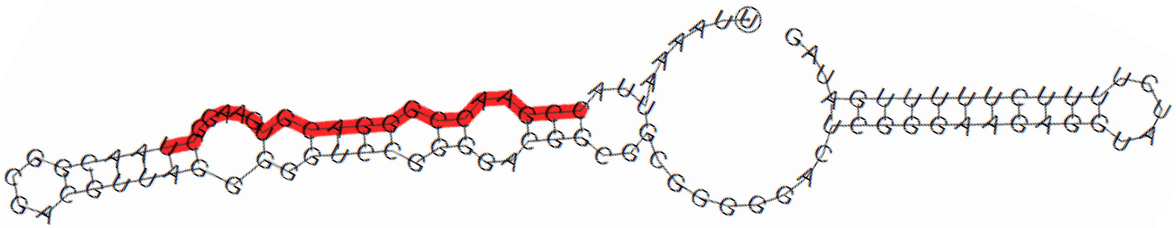

Jcu_MIR403a

```
 1 Jcu_miR403a      100.0%  GGGACATATCTCTAGTTTGTGCGTGAATCGAACCCCATCTTGGCCGTTCATTTTTACGGCCCATCCAATGGTGTTAGATTCACGCATAAACTCGAAATCT
 2 J24_4751762_x1   100.0%  -------------------------------------------------------------------------TTAGATTCACGCATAAACTCGAAA---
 3 J23_807206_x1    100.0%  -------------------------------------------------------------------------TTAGATTCACGCATAAACTCGAA----
 4 J22_2016557_x1   100.0%  -------------------------------------------------------------------------TTAGATTCACGCATAAACTCGA-----
 5 J21_1879371_x66  100.0%  -------------------------------------------------------------------------TTAGATTCACGCATAAACTCG------
 6 J21_2624191_x1   100.0%  --------------------------------------------------------------------------TAGATTCACGCATAAACTCGA-----
 7 J20_214732_x6    100.0%  -------------------------------------------------------------------------TTAGATTCACGCATAAACTC-------
 8 J20_247956_x1    100.0%  --------------------------------------------------------------------------TAGATTCACGCATAAACTCG------
 9 J19_170247_x23   100.0%  -------------------------------------------------------------------------TTAGATTCACGCATAAACT--------
10 J21_1906848_x49  100.0%  ----------TCTAGTTTGTGCGTGAATCGA---------------------------------------------------------------------
11 J18_155840_x1    100.0%  -------------AGTTTGTGCGTGAATCGA---------------------------------------------------------------------
12 J21_1999429_x18  100.0%  ------------TAGTTTGTGCGTGAATCGAAC-------------------------------------------------------------------
13 J21_1907730_x49  100.0%  -------------AGTTTGTGCGTGAATCGAACC------------------------------------------------------------------
14 J24_3498824_x1   100.0%  -------------AGTTTGTGCGTGAATCGAACCCCA---------------------------------------------------------------
15 J20_327318_x1    100.0%  --------------GTTTGTGCGTGAATCGAACC------------------------------------------------------------------
16 J20_218398_x5    100.0%  -------------AGTTTGTGCGTGAATCGAAC-------------------------------------------------------------------
17 J19_187850_x2    100.0%  -------------AGTTTGTGCGTGAATCGAA--------------------------------------------------------------------
```

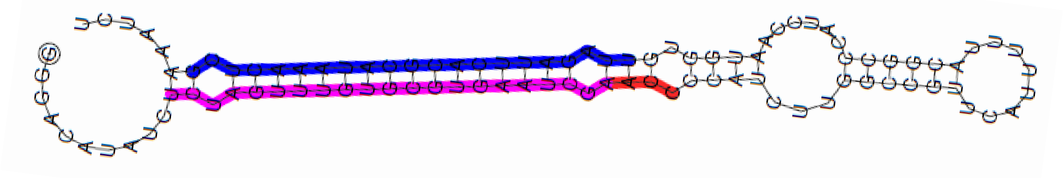

Jcu_MIR403b

```
1 Jcu_miR403b       100.0%  GTGAATCTAATATATAAAATTCGCCAATTTCTATCTATTGGATCGTGTTAGATTCACGCACAAACCCGTAATCTGCCTTTTCAATTTTCCTCGGTTTTGC
2 J21_2372462_x1    100.0%  ---------------------------------------------TGTTAGATTCACGCACAAACC----------------------------------
3 J21_1783288_x157  100.0%  -----------------------------------------------TTAGATTCACGCACAAACCCG--------------------------------
4 J21_2657241_x1    100.0%  ------------------------------------------------TAGATTCACGCACAAACCCGT-------------------------------
5 J19_240308_x1     100.0%  -----------------------------------------------TTAGATTCACGCACAAACC----------------------------------
6 J21_2687580_x1    100.0%  -------------------------------------------------AGATTCACGCACAAACCCGTA------------------------------
7 J21_2100354_x6    100.0%  ---------------------------------------------------ATTCACGCACAAACCCGTAAT----------------------------
```

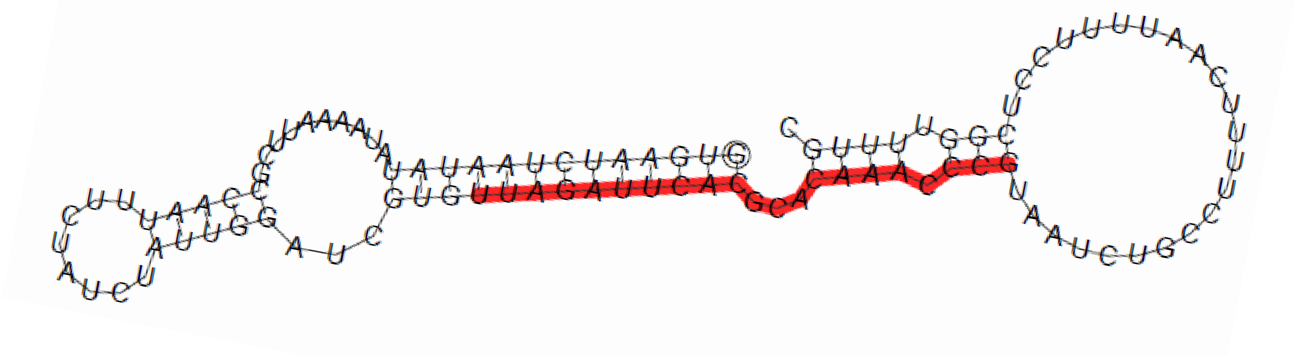

Jcu_MIR472

```
 1 Jcu_miRNA472       100.0%  GAAGGTTCTGGGATGGGTGAGCGGGGAAGATAAATTAATTATGTAAAATTTAATTATTTTTTAATTGTTGTTGTTATTATCTTCCCTACTCCACCCATGCCATAGGTTTCCG
 2 J24_2367131_x2     100.0%  --------------------------------------------------------------------------------CTTCCCTACTCCACCCATGCCATA--------
 3 J23_434998_x6      100.0%  -------------------------------------------------------------------------------TCTTCCCTACTCCACCCATGCCA----------
 4 J22_1276563_x1207  100.0%  -------------------------------------------------------------------------------TCTTCCCTACTCCACCCATGCC-----------
 5 J22_1862328_x2     100.0%  --------------------------------------------------------------------------------CTTCCCTACTCCACCCATGCCA----------
 6 J23_334605_x71     100.0%  ---------------------------------------------------------------------------------TTCCCTACTCCACCCATGCCATA--------
 7 J21_1913014_x46    100.0%  -------------------------------------------------------------------------------TCTTCCCTACTCCACCCATGC------------
 8 J21_2102214_x6     100.0%  --------------------------------------------------------------------------------CTTCCCTACTCCACCCATGCC-----------
 9 J22_1323922_x543   100.0%  ----------------------------------------------------------------------------------TCCCTACTCCACCCATGCCATA--------
10 J21_2262285_x2     100.0%  ---------------------------------------------------------------------------------TTCCCTACTCCACCCATGCCA----------
11 J21_2017235_x15    100.0%  ----------------------------------------------------------------------------------TCCCTACTCCACCCATGCCAT---------
12 J20_358998_x1      100.0%  -------------------------------------------------------------------------------TCTTCCCTACTCCACCCATG-------------
13 J20_230444_x2      100.0%  ----------------------------------------------------------------------------------TCCCTACTCCACCCATGCCA----------
14 J20_214642_x6      100.0%  ---------------------------------------------------------------------------------TTCCCTACTCCACCCATGCC-----------
15 J19_222751_x1      100.0%  -------------------------------------------------------------------------------TCTTCCCTACTCCACCCAT--------------
16 J19_234109_x1      100.0%  ----------------------------------------------------------------------------------TCCCTACTCCACCCATGCC-----------
17 J20_309410_x1      100.0%  ------------------------------------------------------------------------------------CCTACTCCACCCATGCCATA--------
18 J19_204013_x1      100.0%  -------------------------------------------------------------------------------------CTACTCCACCCATGCCATA--------
19 J18_205957_x1      100.0%  -----------------------------------------------------------------------------------CCCTACTCCACCCATGCC-----------
20 J18_224919_x1      100.0%  ----------------------------------------------------------------------------------TCCCTACTCCACCCATGC------------
21 J19_203615_x1      100.0%  -------CTGGGATGGGTGAGCGGGG--------------------------------------------------------------------------------------
22 J21_2135291_x4     100.0%  -------CTGGGATGGGTGAGCGGGGAA------------------------------------------------------------------------------------
23 J20_144959_x458    100.0%  --------TGGGATGGGTGAGCGGGGAA------------------------------------------------------------------------------------
24 J21_1300472_x3397  100.0%  --------TGGGATGGGTGAGCGGGGAAG-----------------------------------------------------------------------------------
25 J22_1382389_x260   100.0%  --------TGGGATGGGTGAGCGGGGAAGA----------------------------------------------------------------------------------
26 J23_542936_x2      100.0%  --------TGGGATGGGTGAGCGGGGAAGAT---------------------------------------------------------------------------------
27 J21_2075015_x8     100.0%  ---------GGGATGGGTGAGCGGGGAAGA----------------------------------------------------------------------------------
28 J20_198874_x22     100.0%  ---------GGGATGGGTGAGCGGGGAAG-----------------------------------------------------------------------------------
29 J19_160483_x68     100.0%  --------TGGGATGGGTGAGCGGGGA-------------------------------------------------------------------------------------
30 J19_218246_x1      100.0%  ---------GGGATGGGTGAGCGGGGAA------------------------------------------------------------------------------------
31 J20_242210_x2      100.0%  ----------GGATGGGTGAGCGGGGAAGA----------------------------------------------------------------------------------
32 J19_266223_x1      100.0%  -----------GATGGGTGAGCGGGGAAGA----------------------------------------------------------------------------------
33 J21_2022247_x14    100.0%  -----------GATGGGTGAGCGGGGAAGATA--------------------------------------------------------------------------------
34 J18_224821_x1      100.0%  ---------GGGATGGGTGAGCGGGGA-------------------------------------------------------------------------------------
35 J18_138345_x9      100.0%  --------TGGGATGGGTGAGCGGGG--------------------------------------------------------------------------------------
36 J18_153102_x1      100.0%  ------------ATGGGTGAGCGGGGAAGA----------------------------------------------------------------------------------
```

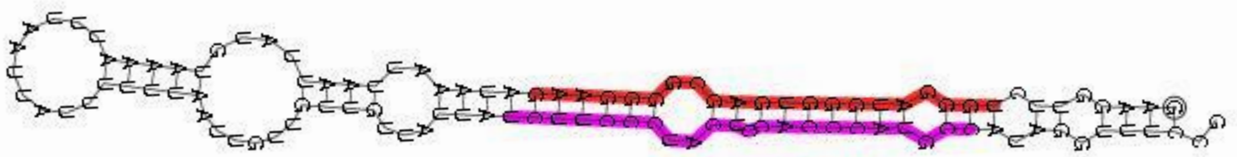

Jcu_MIR482

```
 1 Jcu_miRNA482        100.0%  ATAGAATTCATTGGAAGCTTTTGGCATGGGCGATATGGGCAAGATGAAAATCTCTGTGAATCATCTTACCCACACCCCCCATACCGATGGTTTTCAATAATTCA
 2 J24_640006_x13      100.0%  ---------------------------------------------------------------TCTTACCCACACCCCCCATACCGA-----------------
 3 J23_994672_x1       100.0%  ----------------------------------------------------------------CTTACCCACACCCCCCATACCGA-----------------
 4 J23_583330_x2       100.0%  -----------------------------------------------------------------TTACCCACACCCCCCATACCGAT----------------
 5 J22_1910076_x1      100.0%  ---------------------------------------------------------------TCTTACCCACACCCCCCATACC-------------------
 6 J22_1257953_x1635   100.0%  -----------------------------------------------------------------TTACCCACACCCCCCATACCGA-----------------
 7 J21_2519832_x1      100.0%  ---------------------------------------------------------------TCTTACCCACACCCCCCATAC--------------------
 8 J21_1733438_x237    100.0%  ------------------------------------------------------------------TACCCACACCCCCCATACCGA-----------------
 9 J21_2427790_x1      100.0%  -----------------------------------------------------------------TTACCCACACCCCCCATACCG------------------
10 J20_296679_x1       100.0%  -------------------------------------------------------------------ACCCACACCCCCCATACCGA-----------------
11 J20_222539_x4       100.0%  -----------------------------------------------------------------TTACCCACACCCCCCATACC-------------------
12 J19_269574_x1       100.0%  -----------------------------------------------------------------TTACCCACACCCCCCATAC--------------------
13 J18_150285_x2       100.0%  -----------------------------------------------------------------TTACCCACACCCCCCATA---------------------
14 J18_169283_x1       100.0%  ---------------------------------------------------------------------CCACACCCCCCATACCGA-----------------
18 J18_160042_x1       100.0%  ----------------------GGCATGGGCGATATGGGC----------------------------------------------------------------
19 J19_184544_x3       100.0%  ----------------------GGCATGGGCGATATGGGCA---------------------------------------------------------------
20 J21_2215795_x2      100.0%  ----------------------GGCATGGGCGATATGGGCAAG-------------------------------------------------------------
21 J22_1580795_x44     100.0%  ----------------------GGCATGGGCGATATGGGCAAGA------------------------------------------------------------
22 J24_849914_x10      100.0%  ----------------------GGCATGGGCGATATGGGCAAGATG----------------------------------------------------------
23 J20_362336_x1       100.0%  --------------------------TGGGCGATATGGGCAAGATG----------------------------------------------------------
24 J21_2016920_x15     100.0%  --------------------------TGGGCGATATGGGCAAGATGA---------------------------------------------------------
```

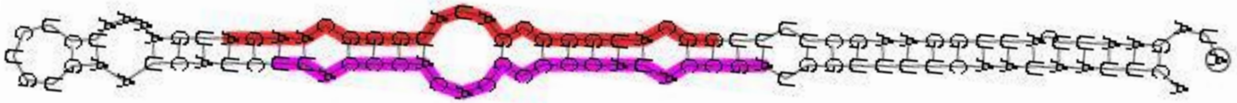

Jcu_MIR535a

```
 1 Jcu_miR535a       100.0%  GAGAATGTGAAGTTCTCGTGCCATCCCCTTCCCTTGTTTGCCTTCAACTTTTGACATGATTGCTTGACGACGAGAGAGAGCACACTGGTTAGCATTCATG
 2 J24_3393844_x1    100.0%  ---------------------------------------------------------------------ACGAGAGAGAGCACACTGGTTAGC-------
 3 J24_1125569_x7    100.0%  ----------------------------------------------------------------------CGAGAGAGAGCACACTGGTTAGCA------
 4 J23_994616_x1     100.0%  ----------------------------------------------------------------------CGAGAGAGAGCACACTGGTTAGC-------
 5 J22_1798553_x4    100.0%  ------------------------------------------------------------------------AGAGAGAGCACACTGGTTAGCA------
 6 J21_2512988_x1    100.0%  ------------------------------------------------------------------------AGAGAGAGCACACTGGTTAGC-------
 7 J22_2117245_x1    100.0%  ---------------------------------------------------------------------ACGAGAGAGAGCACACTGGTTA---------
 8 J20_222235_x4     100.0%  ------------------------------------------------------------------ACGACGAGAGAGAGCACACT--------------
 9 J21_1991208_x20   100.0%  ------------------------------------------------------------------ACGACGAGAGAGAGCACACTG-------------
10 J21_2060390_x9    100.0%  -------------------------------------------------------------------CGACGAGAGAGAGCACACTGG------------
11 J22_1823450_x3    100.0%  -------------------------------------------------------------------CGACGAGAGAGAGCACACTGGT-----------
12 J21_2045407_x11   100.0%  -----------------------------------------------------------TTGCTTGACGACGAGAGAGAG--------------------
13 J21_2684707_x1    100.0%  ------------------------------------------------------------TGCTTGACGACGAGAGAGAGC-------------------
14 J19_172938_x16    100.0%  --------------------------------------------------------------CTTGACGACGAGAGAGAGC-------------------
15 J20_190907_x40    100.0%  --------------------------------------------------------------CTTGACGACGAGAGAGAGCA------------------
16 J21_1730318_x242  100.0%  --------------------------------------------------------------CTTGACGACGAGAGAGAGCAC-----------------
17 J22_2128802_x1    100.0%  --------------------------------------------------------------CTTGACGACGAGAGAGAGCACA----------------
18 J21_1698616_x326  100.0%  ---------------------------------------------------------------TTGACGACGAGAGAGAGCACA----------------
19 J22_1831139_x3    100.0%  ---------------------------------------------------------------TTGACGACGAGAGAGAGCACAC---------------
20 J23_1009530_x1    100.0%  ---------------------------------------------------------------TTGACGACGAGAGAGAGCACACT--------------
21 J22_1560769_x53   100.0%  ----------------------------------------------------------------TGACGACGAGAGAGAGCACACT--------------
22 J21_1882790_x64   100.0%  ----------------------------------------------------------------TGACGACGAGAGAGAGCACAC---------------
23 J20_167862_x159   100.0%  ---------------------------------------------------------------TTGACGACGAGAGAGAGCAC-----------------
24 J20_207631_x11    100.0%  ----------------------------------------------------------------TGACGACGAGAGAGAGCACA----------------
25 J19_182014_x5     100.0%  ----------------------------------------------------------------TGACGACGAGAGAGAGCAC-----------------
26 J19_178478_x7     100.0%  ---------------------------------------------------------------TTGACGACGAGAGAGAGCA------------------
27 J18_134782_x16    100.0%  ----------------------------------------------------------------TGACGACGAGAGAGAGCA------------------
28 J18_130453_x35    100.0%  ---------------------------------------------------------------TTGACGACGAGAGAGAGC-------------------
29 J18_145769_x3     100.0%  --------------------------------------------------------------CTTGACGACGAGAGAGAG--------------------
```

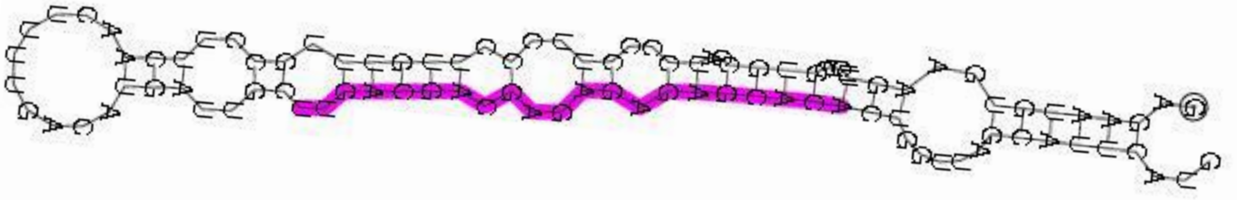

Jcu_MIR2022

```
1 Jcu_miR2022      100.0%  TGTATAGGTGGCAAAAGCAATTAGTTAAAAGCAATTTTAATTTGATCCTTGAACCATCTTTTGTGGAATTTAATATTTGATACTGTGAATTGTTCGTCTGTCTAGAAATCGCTCAAACCGGATGGAATTGTTTCGATTTTCTCTAAATTATTCAATTAATACTTGAACTAGTTGCTTTTGCCCCCTACACCATGTGG
2 J23_652814_x1    100.0%  -------------------------------------------------------------------------------------------------------------------------------------------------------------------TGAACTAGTTGCTTTTGCCCCCT-----------
3 J21_2534057_x1   100.0%  ---------------------------------------------------------------------------------------------------------------------------------------------------------------------AACTAGTTGCTTTTGCCCCCT-----------
4 J21_2458317_x1   100.0%  ------------------------------------------------------------------------------------------------------------------------------------------------------------------------TAGTTGCTTTTGCCCCCTACA--------
5 J21_2605918_x1   100.0%  --TATAGGTGGCAAAAGCAATTA------------------------------------------------------------------------------------------------------------------------------------------------------------------------------
6 J21_1867235_x75  100.0%  ---ATAGGTGGCAAAAGCAATTAG-------------------------------------------------------------------------------------------------------------------------------------------------------------------------
7 J18_223069_x1    100.0%  ------GGTGGCAAAAGCAATTAG-------------------------------------------------------------------------------------------------------------------------------------------------------------------------
```

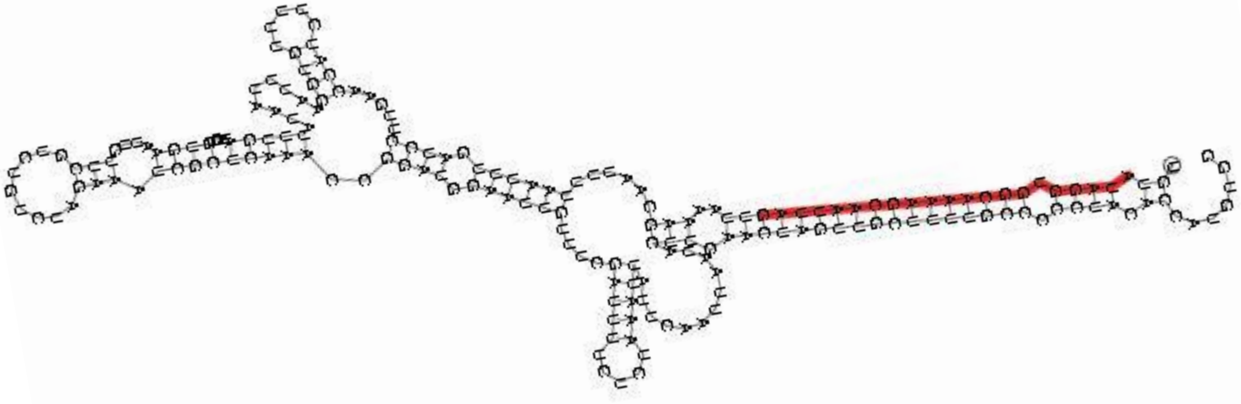

Jcu_MIR2911

```
 1 Jcu_miR2911a    100.0%  TCGGCGGACTGCTCGAGCTGCTCCCGCGGCGAGAGCGGGTCGCCGCGTGCCGGCCGGGGGGCGGACTGGGAACGGGCCTTCCGGGGGGCCTCCCCCGGCG
 2 J25_105432_x2   100.0%  ----------------------------------------CGCCGCGTGCCGGCCGGGGGGCGGA-----------------------------------
 3 J23_618322_x2   100.0%  ------------------------------------------CCGCGTGCCGGCCGGGGGGCGGA-----------------------------------
 4 J24_4571697_x1  100.0%  -------------------------------------------CGCGTGCCGGCCGGGGGGCGGACT---------------------------------
 5 J22_2075064_x1  100.0%  --------------------------------------------GCGTGCCGGCCGGGGGGCGGAC----------------------------------
 6 J22_2144406_x1  100.0%  ---------------------------------------TCGCCGCGTGCCGGCCGGGGGG---------------------------------------
 7 J22_1824305_x3  100.0%  ------------------------------------------------GCCGGCCGGGGGGCGGACTGGG------------------------------
 8 J23_342137_x49  100.0%  ------------------------------------------------GCCGGCCGGGGGGCGGACTGGGA-----------------------------
 9 J24_1380623_x5  100.0%  ------------------------------------------------GCCGGCCGGGGGGCGGACTGGGAA----------------------------
10 J22_1868036_x2  100.0%  -------------------------------------------------CCGGCCGGGGGGCGGACTGGGA-----------------------------
11 J18_152301_x2   100.0%  -----------------------------------------------TGCCGGCCGGGGGGCGGA-----------------------------------
12 J18_217876_x1   100.0%  -----------------------------------------------------CCGGGGGGCGGACTGGGA-----------------------------
13 J18_180736_x1   100.0%  --------------------------------------------------------GGGGGCGGACTGGGAACG--------------------------
14 J24_4858065_x1  100.0%  --------------------------------------------------------------GGACTGGGAACGGGCCTTCCGGGG--------------
```

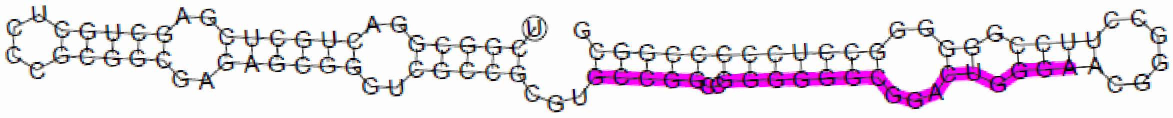

Jcu_MIR2911

```
 1 Jcu_miR2911a    100.0%  TCGGCGGACTGCTCGAGCTGCTCCCGCGGCGAGAGCGGGTCGCCGCGTGCCGGCCGGGGGGCGGACTGGGAACGGGCCTTCCGGGGGGCCTCCCCCGGCG
 2 J25_105432_x2   100.0%  ----------------------------------------CGCCGCGTGCCGGCCGGGGGGCGGA-----------------------------------
 3 J23_618322_x2   100.0%  ------------------------------------------CCGCGTGCCGGCCGGGGGGCGGA-----------------------------------
 4 J24_4571697_x1  100.0%  -------------------------------------------CGCGTGCCGGCCGGGGGGCGGACT---------------------------------
 5 J22_2075064_x1  100.0%  --------------------------------------------GCGTGCCGGCCGGGGGGCGGAC----------------------------------
 6 J22_2144406_x1  100.0%  ---------------------------------------TCGCCGCGTGCCGGCCGGGGGG---------------------------------------
 7 J22_1824305_x3  100.0%  ------------------------------------------------GCCGGCCGGGGGGCGGACTGGG------------------------------
 8 J23_342137_x49  100.0%  ------------------------------------------------GCCGGCCGGGGGGCGGACTGGGA-----------------------------
 9 J24_1380623_x5  100.0%  ------------------------------------------------GCCGGCCGGGGGGCGGACTGGGAA----------------------------
10 J22_1868036_x2  100.0%  -------------------------------------------------CCGGCCGGGGGGCGGACTGGGA-----------------------------
11 J18_152301_x2   100.0%  -----------------------------------------------TGCCGGCCGGGGGGCGGA-----------------------------------
12 J18_217876_x1   100.0%  -----------------------------------------------------CCGGGGGGCGGACTGGGA-----------------------------
13 J18_180736_x1   100.0%  --------------------------------------------------------GGGGGCGGACTGGGAACG--------------------------
14 J24_4858065_x1  100.0%  --------------------------------------------------------------GGACTGGGAACGGGCCTTCCGGGG--------------
```


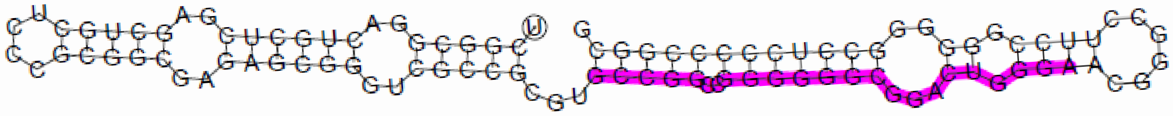

Supplement: Figure S2 — Predicted secondary structures of known miRNA precursors in J. curcas . Locations and expressions of small RNAs mapped onto these precursors are presented. Read sequences corresponding to miRNA candidates, which are located in the 5p and 3p arms and labeled in red and purple, respectively. Values on the left side of the miRNA sequences represent the miRNA length (Jn) and read counts (x n) in the mature seed library. (PDF) [file pone.0083727.s002.pdf]
