# Supplementary material for: Identifying MicroRNAs and Transcript Targets in Jatropha Seeds
Source: PLoS One. 2014 Feb 13;9(2):e83727. doi: 10.1371/journal.pone.0083727 (PMC3923737; doi:10.1371/journal.pone.0083727)

Figure S3. Predicted secondary structures of novel miRNA precursors in *J. curcas*. The locations and the expression of small RNAs mapped onto these precursors are shown here. The sequences of miRNA candidates located in the 5p and 3p arms are labeled in red and purple, respectively. Values on the left side of the miRNA sequence represent miRNA length (Jn) and read counts (x n) in the mature seed library.

Jcu_nMIR001

1	Jcu_nmiR001	100.0%	CTCCGTCCCTCTGGGCACGTTGTTCATTCCTCTTCCTAAAATGGTTTCTCTTTTTTTCTCTGTATTGTATTTTACATATATTGAATCAAGCGAATCCATTTTAGGAAGAGAATGAATACCGTTATCATAGAGATACTGAAAC
2	J24_1490948_x5	100.0%	TTCATTCCTCTTCCTAAAATGGTT
3	J23_361609_x24	100.0%	TTCATTCCTCTTCCTAAAATGGT
4	J22_1171415_x11899	100.0%	TTCATTCCTCTTCCTAAAATGG
5	J22_1737028_x9	100.0%	CATTCCTCTTCCTAAAATGGTT
6	J21_1869386_x73	100.0%	TTCATTCCTCTTCCTAAAATG
7	J21_1908746_x48	100.0%	TCATTCCTCTTCCTAAAATGG
8	J21_2135003_x4	100.0%	ATTCCTCTTCCTAAAATGGTT
9	J22_1618311_x32	100.0%	ATTCCTCTTCCTAAAATGGTTT
10	J20_207741_x11	100.0%	TTCATTCCTCTTCCTAAAAT
11	J20_218218_x5	100.0%	CATTCCTCTTCCTAAAATGG
12	J22_2047378_x1	100.0%	TTCCTCTTCCTAAAATGGTTTC
13	J19_170339_x23	100.0%	TTCATTCCTCTTCCTAAAA
14	J20_350497_x1	100.0%	TCCTCTTCCTAAAATGGTTT
15	J19_177125_x9	100.0%	ATTCCTCTTCCTAAAATGG
16	J18_136819_x12	100.0%	TTCATTCCTCTTCCTAAA
17	J18_138048_x9	100.0%	TTCCTCTTCCTAAAATGG
18	J19_254766_x1	100.0%	CCTCTTCCTAAAATGGTTT
19	J21_2283031_x1	100.0%	CCTCTTCCTAAAATGGTTTCT
21	J21_2322267_x1	100.0%	TTCCTAAAATGGTTTCTCTTT
22	J21_2038028_x12	100.0%	TCCTAAAATGGTTTCTCTTTT
23	J19_233819_x1	100.0%	CTAAAATGGTTTCTCTTTT
24	J21_2136083_x4	100.0%	CTAAAATGGTTTCTCTTTTTT
28	J21_2444573_x1	100.0%	AATCAAGCGAATCCATTTTAG
29	J20_227994_x3	100.0%	TCAAGCGAATCCATTTTAGG
30	J21_1834242_x100	100.0%	TCAAGCGAATCCATTTTAGGA
31	J20_206655_x12	100.0%	CAAGCGAATCCATTTTAGGA
32	J21_1967426_x27	100.0%	CAAGCGAATCCATTTTAGGAA
33	J19_238660_x1	100.0%	CAAGCGAATCCATTTTAGG
34	J19_230927_x1	100.0%	AAGCGAATCCATTTTAGGA
35	J19_273733_x1	100.0%	AATCCATTTTAGGAAGAGA
36	J21_2246617_x2	100.0%	AATCCATTTTAGGAAGAGAAT
37	J20_246202_x1	100.0%	ATCCATTTTAGGAAGAGAAT
38	J21_2400367_x1	100.0%	ATCCATTTTAGGAAGAGAATG
39	J23_1070910_x1	100.0%	ATCCATTTTAGGAAGAGAATGAA
40	J23_792043_x1	100.0%	TCCATTTTAGGAAGAGAATGAAT
41	J24_1493318_x5	100.0%	TCCATTTTAGGAAGAGAATGAATA
42	J22_1787069_x5	100.0%	CCATTTTAGGAAGAGAATGAAT
43	J21_2194843_x2	100.0%	TCCATTTTAGGAAGAGAATGA
44	J21_1577263_x791	100.0%	CCATTTTAGGAAGAGAATGAA
45	J20_209641_x10	100.0%	CATTTTAGGAAGAGAATGAA
46	J20_225033_x3	100.0%	CCATTTTAGGAAGAGAATGA
47	J20_200147_x20	100.0%	ATTTTAGGAAGAGAATGAAT
48	J22_1802005_x4	100.0%	CATTTTAGGAAGAGAATGAATA
49	J21_1341227_x2905	100.0%	ATTTTAGGAAGAGAATGAATA
50	J22_1936494_x1	100.0%	ATTTTAGGAAGAGAATGAATAC
51	J21_2051121_x10	100.0%	TTTTAGGAAGAGAATGAATAC
52	J21_1880542_x65	100.0%	TTTAGGAAGAGAATGAATACC
53	J20_197507_x24	100.0%	TTAGGAAGAGAATGAATACC
54	J20_147159_x426	100.0%	TTTTAGGAAGAGAATGAATA
55	J19_180799_x5	100.0%	ATTTTAGGAAGAGAATGAA
56	J18_142369_x4	100.0%	ATTTTAGGAAGAGAATGA
57	J19_196459_x1	100.0%	TTAGGAAGAGAATGAATAC
58	J21_2641590_x1	100.0%	TTAGGAAGAGAATGAATACCG
59	J23_768545_x1	100.0%	TTAGGAAGAGAATGAATACCGTT
60	J19_193066_x2	100.0%	TTTAGGAAGAGAATGAATA
61	J18_135778_x14	100.0%	TTAGGAAGAGAATGAATA
62	J19_239914_x1	100.0%	GGAAGAGAATGAATACCGT

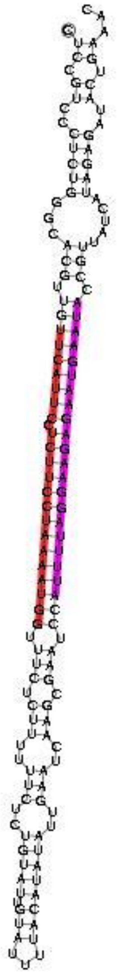

Jcu_nMIR002

```
 1 Jcu_nmiR002        100.0%   GGAGTATGTTAGTTTGTACTGGAGTGACCAACCTTCTTCCCTTCAGATTATGGATGGACGTTGGTCACTCCATTACATGCCAACATCAATTCCA
 2 J24_1590668_x4     100.0%   ----------------TACTGGAGTGACCAACCTTCTTCC------------------------------------------------------
 3 J23_410601_x8      100.0%   --------------TGTACTGGAGTGACCAACCTTCT---------------------------------------------------------
 4 J22_1711335_x13    100.0%   --------------TGTACTGGAGTGACCAACCTTC----------------------------------------------------------
 5 J21_2278662_x1     100.0%   ----------------TACTGGAGTGACCAACCTTCT---------------------------------------------------------
 6 J21_1218924_x4871  100.0%   --------------TGTACTGGAGTGACCAACCTT-----------------------------------------------------------
 7 J20_196181_x28     100.0%   ---------------GTACTGGAGTGACCAACCTT-----------------------------------------------------------
 8 J20_166183_x178    100.0%   --------------TGTACTGGAGTGACCAACCT------------------------------------------------------------
 9 J21_2629934_x1     100.0%   ------------TTTGTACTGGAGTGACCAACC-------------------------------------------------------------
10 J20_226785_x3      100.0%   -------------TTGTACTGGAGTGACCAACC-------------------------------------------------------------
11 J19_170224_x23     100.0%   --------------TGTACTGGAGTGACCAACC-------------------------------------------------------------
12 J18_151293_x2      100.0%   ---------------GTACTGGAGTGACCAACC-------------------------------------------------------------
13 J18_130208_x35     100.0%   --------------TGTACTGGAGTGACCAAC--------------------------------------------------------------
14 J21_1791174_x146   100.0%   ------TGTTAGTTTGTACTGGAGTGA-------------------------------------------------------------------
15 J21_2531708_x1     100.0%   --------TTAGTTTGTACTGGAGTGACC-----------------------------------------------------------------
16 J21_2013346_x16    100.0%   ----------------------------------------------------------CGTTGGTCACTCCATTACATG---------------
17 J19_211847_x1      100.0%   ------------------------------------------------------------TTGGTCACTCCATTACATG---------------
18 J20_348924_x1      100.0%   ------------------------------------------------------------TTGGTCACTCCATTACATGC--------------
```

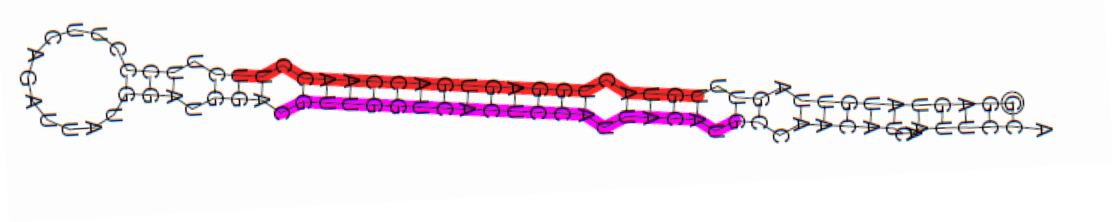

Jcu_nMIR003

```
 1 Jcu_nmiR003        100.0%   AAGAAGGGAAAGGTTTTGGACGCAACTTGGAGTGGCACCATTAACACTTGGATAGGGTGGGAGTAAATGGTGCCACGCTGAGTGCGTCTAAAATACTTGCCCTTT
 2 J25_263445_x1      100.0%   ------------GTTTTGGACGCAACTTGGAGTGGCA--------------------------------------------------------------------
 3 J25_151515_x1      100.0%   --------------TTTGGACGCAACTTGGAGTGGCACC------------------------------------------------------------------
 4 J22_1848618_x2     100.0%   -------------TTTTGGACGCAACTTGGAGTGG----------------------------------------------------------------------
 5 J22_1790169_x5     100.0%   --------------TTTGGACGCAACTTGGAGTGGC---------------------------------------------------------------------
 6 J19_179638_x6      100.0%   --------------------CGCAACTTGGAGTGGCACC------------------------------------------------------------------
 7 J22_1306546_x680   100.0%   --------------------CGCAACTTGGAGTGGCACCATT---------------------------------------------------------------
 8 J21_2084322_x7     100.0%   ---------------------GCAACTTGGAGTGGCACCATT---------------------------------------------------------------
 9 J20_337129_x1      100.0%   -------------------------------------------------------------------TGGTGCCACGCTGAGTGCGT------------------
10 J21_1873402_x70    100.0%   -------------------------------------------------------------------TGGTGCCACGCTGAGTGCGTC-----------------
11 J18_194143_x1      100.0%   ----------------------------------------------------------------------TGCCACGCTGAGTGCGTC-----------------
```

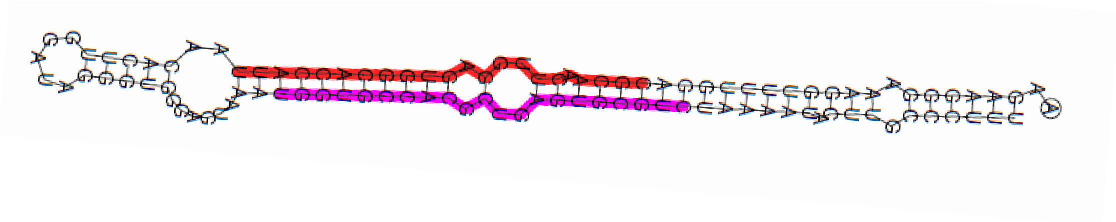

Jcu_nMIR004

```
 1 Jcu_nmiR004      100.0%  GGATGGCGCGCCTAGCACTCCATGTCCATGGCTCACCAGAACCCCCAAAGGTGCAAGCTGGGCATGGAGTGGAAGGCGGTCGATTTCCT
 2 J24_105665_x118  100.0%  ----------------ACTCCATGTCCATGGCTCACCAGA-------------------------------------------------
 3 J24_2017739_x3   100.0%  -----------------CTCCATGTCCATGGCTCACCAGAA------------------------------------------------
 4 J23_328607_x105  100.0%  -----------------CTCCATGTCCATGGCTCACCAGA-------------------------------------------------
 5 J22_1835948_x3   100.0%  ------------------TCCATGTCCATGGCTCACCAGA-------------------------------------------------
 6 J21_2091630_x6   100.0%  -----------------CTCCATGTCCATGGCTCACCA---------------------------------------------------
 7 J21_2512836_x1   100.0%  -------------------CCATGTCCATGGCTCACCAGA-------------------------------------------------
 8 J20_265270_x1    100.0%  -----------------CTCCATGTCCATGGCTCACC----------------------------------------------------
 9 J20_238074_x2    100.0%  ------------------TCCATGTCCATGGCTCACCA---------------------------------------------------
10 J20_337313_x1    100.0%  --------------------CATGTCCATGGCTCACCAGA-------------------------------------------------
11 J19_190042_x2    100.0%  ---------------------ATGTCCATGGCTCACCAGA-------------------------------------------------
12 J18_178922_x1    100.0%  -----------------------GTCCATGGCTCACCAGAA------------------------------------------------
```

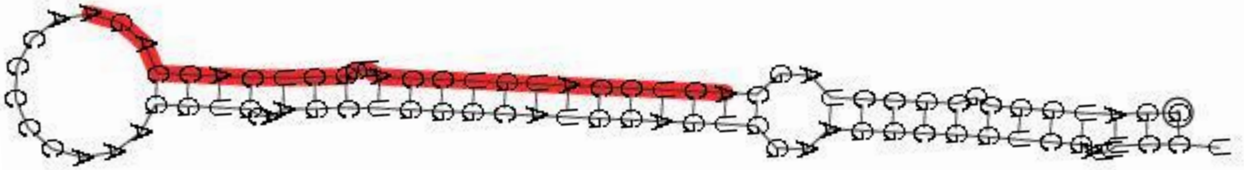

Jcu_nMIR005

```
1 Jcu_nmiR005     100.0%  GCAAGTGCTTTCTAATATCTGCTTGATATTTTTATTTTCACAGTTGCTTTCTGAAACAGGTATGAAATTGACAAGGTCACGGCTGGGGAGCGATTGGGGT
2 J21_2226389_x2  100.0%  -----------------------------------------------------------GTATGAAATTGACAAGGTCAC--------------------
3 J21_1950470_x31 100.0%  --------------------------------------------------------------TGAAATTGACAAGGTCACGGC-----------------
4 J21_2425991_x1  100.0%  ---------------------------------------------------------------GAAATTGACAAGGTCACGGCT----------------
5 J18_175705_x1   100.0%  -----------------------------------------------------------GTATGAAATTGACAAGGT-----------------------
6 J19_193116_x2   100.0%  -----------------------------------------------------------GTATGAAATTGACAAGGTC----------------------
```

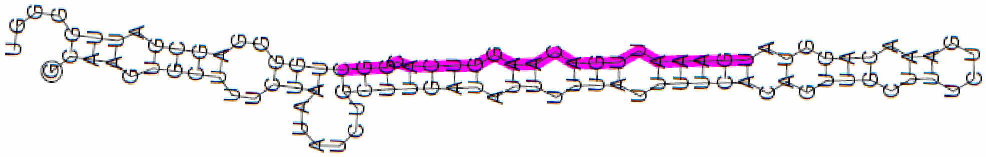

Jcu_nMIR006

```
 1 Jcu_nmiR006      100.0%   GGGAAATGCCCAGCTTGAGAATCGGATGCCCCCGGCGTCCGGATTTGAGGCTGGGGAAGCGGCCCCCGCGGCCGGCCCGGGCCCCGGCCCCCCGCAGGGG
 2 J25_65447_x5     100.0%   -GGAAATGCCCAGCTTGAGAATCGGA--------------------------------------------------------------------------
 3 J25_253655_x1    100.0%   --GAAATGCCCAGCTTGAGAATCGGAT-------------------------------------------------------------------------
 4 J24_2572547_x2   100.0%   --GAAATGCCCAGCTTGAGAATCGGA--------------------------------------------------------------------------
 5 J24_1033433_x8   100.0%   ---AAATGCCCAGCTTGAGAATCGGAT-------------------------------------------------------------------------
 6 J25_261305_x1    100.0%   ----AATGCCCAGCTTGAGAATCGGATGC-----------------------------------------------------------------------
 7 J23_589822_x2    100.0%   ---AAATGCCCAGCTTGAGAATCGGA--------------------------------------------------------------------------
 8 J23_553912_x2    100.0%   ----AATGCCCAGCTTGAGAATCGGAT-------------------------------------------------------------------------
 9 J24_1282614_x6   100.0%   -----ATGCCCAGCTTGAGAATCGGATGC-----------------------------------------------------------------------
10 J25_66842_x5     100.0%   -----ATGCCCAGCTTGAGAATCGGATGCC----------------------------------------------------------------------
11 J24_2056238_x3   100.0%   ------TGCCCAGCTTGAGAATCGGATGCC----------------------------------------------------------------------
12 J25_62979_x6     100.0%   ------TGCCCAGCTTGAGAATCGGATGCCC---------------------------------------------------------------------
13 J22_1737757_x9   100.0%   ----AATGCCCAGCTTGAGAATCGGA--------------------------------------------------------------------------
14 J22_1762767_x7   100.0%   -----ATGCCCAGCTTGAGAATCGGAT-------------------------------------------------------------------------
15 J21_1958330_x29  100.0%   -----ATGCCCAGCTTGAGAATCGGA--------------------------------------------------------------------------
16 J22_1883552_x2   100.0%   ------TGCCCAGCTTGAGAATCGGATG------------------------------------------------------------------------
17 J21_1983808_x22  100.0%   ------TGCCCAGCTTGAGAATCGGAT-------------------------------------------------------------------------
18 J20_190043_x42   100.0%   ------TGCCCAGCTTGAGAATCGGA--------------------------------------------------------------------------
19 J25_100422_x2    100.0%   --------CCCAGCTTGAGAATCGGATGCCCCC-------------------------------------------------------------------
20 J24_3014937_x2   100.0%   ---------CCAGCTTGAGAATCGGATGCCCCC-------------------------------------------------------------------
21 J23_520532_x3    100.0%   ----------CAGCTTGAGAATCGGATGCCCCC-------------------------------------------------------------------
22 J25_92662_x2     100.0%   ----------CAGCTTGAGAATCGGATGCCCCCGG-----------------------------------------------------------------
23 J24_2454469_x2   100.0%   -----------AGCTTGAGAATCGGATGCCCCCGG-----------------------------------------------------------------
24 J25_108106_x2    100.0%   -----------AGCTTGAGAATCGGATGCCCCCGGC----------------------------------------------------------------
25 J24_1552192_x4   100.0%   ------------GCTTGAGAATCGGATGCCCCCGGC----------------------------------------------------------------
26 J25_117204_x1    100.0%   ------------GCTTGAGAATCGGATGCCCCCGGCG---------------------------------------------------------------
27 J24_3786175_x1   100.0%   -------------CTTGAGAATCGGATGCCCCCGGCG---------------------------------------------------------------
28 J25_232027_x1    100.0%   -------------CTTGAGAATCGGATGCCCCCGGCGT--------------------------------------------------------------
29 J24_5024215_x1   100.0%   --------------TTGAGAATCGGATGCCCCCGGCGT--------------------------------------------------------------
30 J23_426198_x7    100.0%   -------------CTTGAGAATCGGATGCCCCCGGC----------------------------------------------------------------
31 J23_431494_x6    100.0%   --------------TTGAGAATCGGATGCCCCCGGCG---------------------------------------------------------------
32 J22_1823393_x3   100.0%   -------------CTTGAGAATCGGATGCCCCCGG-----------------------------------------------------------------
33 J22_1848222_x2   100.0%   --------------TTGAGAATCGGATGCCCCCGGC----------------------------------------------------------------
34 J24_4000166_x1   100.0%   ---------------TGAGAATCGGATGCCCCCGGCGTC-------------------------------------------------------------
35 J21_2133295_x4   100.0%   ------------GCTTGAGAATCGGATGCCCCC-------------------------------------------------------------------
36 J21_2515166_x1   100.0%   -------------CTTGAGAATCGGATGCCCCCG------------------------------------------------------------------
37 J20_213322_x7    100.0%   -------------CTTGAGAATCGGATGCCCCC-------------------------------------------------------------------
38 J21_2482950_x1   100.0%   ---------------TGAGAATCGGATGCCCCCGGC----------------------------------------------------------------
39 J21_2621048_x1   100.0%   ----------------GAGAATCGGATGCCCCCGGCG---------------------------------------------------------------
40 J20_238776_x2    100.0%   -----------------AGAATCGGATGCCCCCGGCG---------------------------------------------------------------
41 J19_179908_x6    100.0%   --------------TTGAGAATCGGATGCCCCC-------------------------------------------------------------------
42 J18_148267_x2    100.0%   ---------------TGAGAATCGGATGCCCCC-------------------------------------------------------------------
43 J19_266257_x1    100.0%   ------------------GAATCGGATGCCCCCGGCG---------------------------------------------------------------
44 J19_255680_x1    100.0%   --------------------ATCGGATGCCCCCGGCGTC-------------------------------------------------------------
45 J21_2455298_x1   100.0%   --------------------ATCGGATGCCCCCGGCGTCCG-----------------------------------------------------------
```

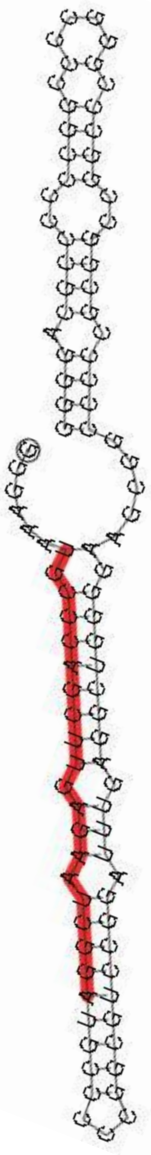

Jcu_nMIR007

```
 1 Jcu_nmiR007     100.0%  TTGTTGTTGGTAAATTTCATTAATCTCCATTCCTTAGGATCAACGCAGTCCAATAACTGACCCTAAGAATGGAGATTAATCAAATTTATCGAT
 2 J25_161106_x1   100.0%  --------------------TAATCTCCATTCCTTAGGATCAACG------------------------------------------------
 3 J23_851482_x1   100.0%  -----------------CATTAATCTCCATTCCTTAGGAT-----------------------------------------------------
 4 J22_1490378_x98 100.0%  -------------------TTAATCTCCATTCCTTAGGATC----------------------------------------------------
 5 J22_1529228_x69 100.0%  -----------------CATTAATCTCCATTCCTTAGGA------------------------------------------------------
 6 J21_2134079_x4  100.0%  -------------------TTAATCTCCATTCCTTAGGAT-----------------------------------------------------
 7 J21_2172690_x3  100.0%  --------------------TAATCTCCATTCCTTAGGATC----------------------------------------------------
 8 J21_2618456_x1  100.0%  -----------------CATTAATCTCCATTCCTTAGG-------------------------------------------------------
 9 J21_2386394_x1  100.0%  ------------------ATTAATCTCCATTCCTTAGGA------------------------------------------------------
10 J20_344906_x1   100.0%  -------------------TTAATCTCCATTCCTTAGGA------------------------------------------------------
11 J19_277473_x1   100.0%  ---------------------AATCTCCATTCCTTAGGAT-----------------------------------------------------
12 J19_209882_x1   100.0%  ---------------------------CATTCCTTAGGATCAACGC-----------------------------------------------
13 J21_2422668_x1  100.0%  ------------------------------------------------------AACTGACCCTAAGAATGGAGA------------------
14 J19_199841_x1   100.0%  --------------------------------------------------------CTGACCCTAAGAATGGAGA------------------
15 J18_218165_x1   100.0%  -------------------------------------------------------ACTGACCCTAAGAATGGA--------------------
16 J20_241712_x2   100.0%  -----------------------------------------------------------ACCCTAAGAATGGAGATTAA--------------
17 J20_221583_x4   100.0%  ------------------------------------------------------------CCCTAAGAATGGAGATTAAT-------------
18 J21_2353966_x1  100.0%  ------------------------------------------------------------CCCTAAGAATGGAGATTAATC------------
19 J19_246477_x1   100.0%  -------------------------------------------------------------CCTAAGAATGGAGATTAAT-------------
20 J20_188905_x45  100.0%  --------------------------------------------------------------CTAAGAATGGAGATTAATCA-----------
21 J21_1979352_x23 100.0%  --------------------------------------------------------------CTAAGAATGGAGATTAATCAA----------
22 J22_2086594_x1  100.0%  --------------------------------------------------------------CTAAGAATGGAGATTAATCAAA---------
23 J21_2404219_x1  100.0%  ---------------------------------------------------------------TAAGAATGGAGATTAATCAAA---------
24 J18_180985_x1   100.0%  ------------------------------------------------------------CCCTAAGAATGGAGATTA---------------
```

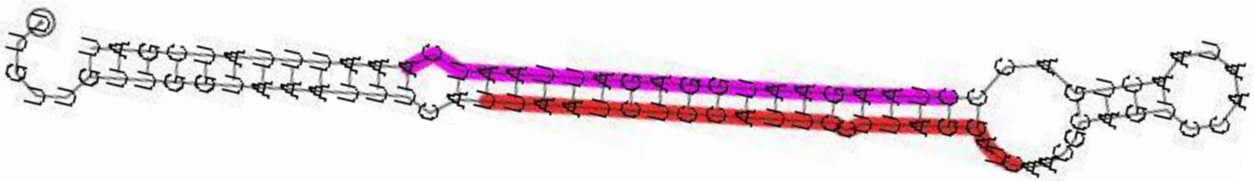

Jcu_nMIR008

```
1 Jcu_nmiR008      100.0% GTGTGGTTCCATTCCCAGAATCACACTGTATAGGTGGCAAAAGCAATTAGTTAAAAGCAATTTTAATTTGATCCTTGATAGAAATCGCTCACCATCTTTTGTGGAATTTAATATTTGATACTGTGAATTG'CGTCTGTCAAACCGGATGGAATTGTTTCGATTTTCTCTAAATTATTCAATTAATACTTGAACTAGTTGCTTTTGCCCCCTACAC
2 J23_652814_x1    100.0% -------------------------------------------------------------------------------------------------------------------------------------------------------------------------------------------TGAACTAGTTGCTTTTGCCCCCT----
3 J21_2534057_x1   100.0% ---------------------------------------------------------------------------------------------------------------------------------------------------------------------------------------------AACTAGTTGCTTTTGCCCCCT----
4 J21_2458317_x1   100.0% ------------------------------------------------------------------------------------------------------------------------------------------------------------------------------------------------TAGTTGCTTTTGCCCCCTACA-
5 J21_2605918_x1   100.0% ----------------------------TATAGGTGGCAAAAGCAATTA---------------------------------------------------------------------------------------------------------------------------------------------------------------------
6 J21_1867235_x75  100.0% -----------------------------ATAGGTGGCAAAAGCAATTAG--------------------------------------------------------------------------------------------------------------------------------------------------------------------
7 J18 223069 x1    100.0% --------------------------------GGTGGCAAAAGCAATTAG--------------------------------------------------------------------------------------------------------------------------------------------------------------------
```

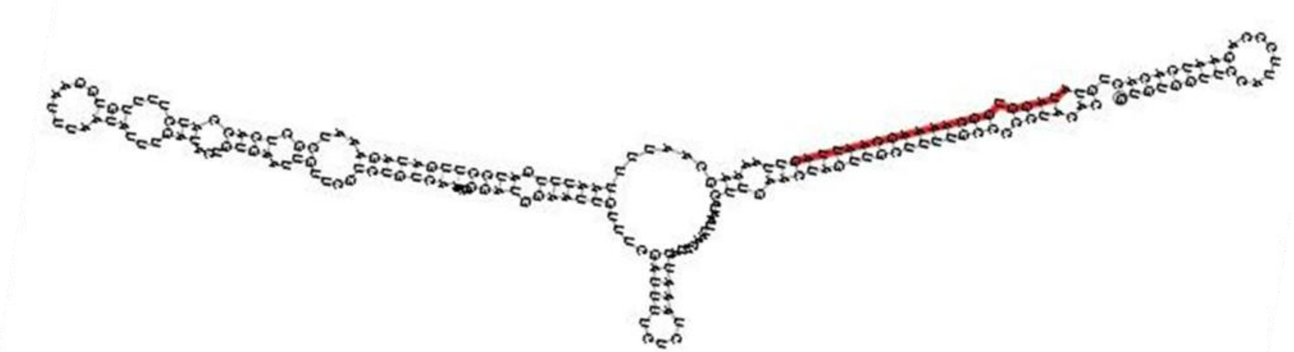

Jcu_nMIR009

```
1 Jcu_nmiR009      100.0% CAGTAAGTGAAGGTAGATTTAGTCATTGTGAAGCATCTTGTTGCTTCTAGAACTTTTGTTGAGGTTTCCATATGGAATATAAACCTCTACCAAAAATTTAAACTCAATGAGATGCTTCACTATGATTGCATCTACTTTCATTTACTAAA
2 J22_1841414_x2   100.0% -------TGAAGGTAGATTTAGTCATTGT------------------------------------------------------------------------------------------------------------------------
3 J21_1876435_x68  100.0% -------TGAAGGTAGATTTAGTCATTG-------------------------------------------------------------------------------------------------------------------------
4 J20_375353_x1    100.0% -------TGAAGGTAGATTTAGTCATT--------------------------------------------------------------------------------------------------------------------------
5 J21_2226203_x2   100.0% -----------------TTTAGTCATTGTGAAGCATCT---------------------------------------------------------------------------------------------------------------
6 J22_1890998_x1   100.0% ------------------TTAGTCATTGTGAAGCATCTTG-------------------------------------------------------------------------------------------------------------
7 J21 2344384 x1   100.0% ------------------------ATTGTGAAGCATCTTGTTGCT--------------------------------------------------------------------------------------------------------
```

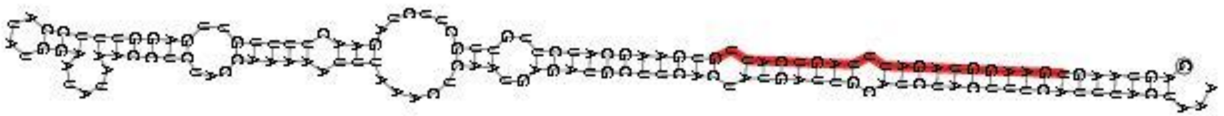

Jcu_nMIR010

```
1 Jcu_nmiR010      100.0%  TGTAGATTCCCTGTTGTTTGACCACTTAGATTGGAGTTTCCCTGTTGACTGGAAGCTGTTATTTTGAATTTTAACTGAAAAGTTGAGAACATTATCACCATTTCTTTTTGTTGAAGTTCAAAACAATAGCTTCCAATCAACAGAGCAACTCCTAACTAAATGGCTAAATAACATGAAATTTACAT
4 J21_1893870_x57  100.0%  ----------------------------------------CCTGTTGACTGGAAGCTGTTA----------------------------------------------------------------------------------------------------------------------------
5 J20_282631_x1    100.0%  -----------------------------------------CTGTTGACTGGAAGCTGTTA----------------------------------------------------------------------------------------------------------------------------
6 J21 2433294 x1   100.0%  ------------------------------------------------CTGGAAGCTGTTATTTTGAAT--------------------------------------------------------------------------------------------------------------------
```

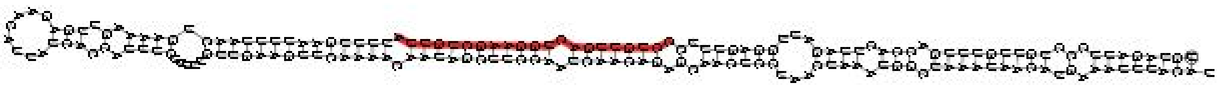

Jcu_nMIR011

```
1 Jcu_nmiR011      100.0%  AAACTCGATTTACATGTGAACGAGCTTGTCAGCCATTAGTTTTAAAATTATACTACGATCCCAATAAAATCTTAAATCTAATAGCTGACAAGCTCGTTCACATATAAGATCGAGCTATATATATACATATATATATACCCATAGCTTC
2 J21_1942741_x34  100.0%  ------------CATGTGAACGAGCTTGTCAGC-------------------------------------------------------------------------------------------------------------------
3 J20_282394_x1    100.0%  -------------ATGTGAACGAGCTTGTCAGC-------------------------------------------------------------------------------------------------------------------
4 J21_2139383_x4   100.0%  ------------------AACGAGCTTGTCAGCCATTAG-------------------------------------------------------------------------------------------------------------
5 J21_2292001_x1   100.0%  ------------------------------------------------------------------------------TAATAGCTGACAAGCTCGTTC-------------------------------------------------
6 J21_2160216_x3   100.0%  -------------------------------------------------------------------------------AATAGCTGACAAGCTCGTTCA------------------------------------------------
7 J20_272095_x1    100.0%  --------------------------------------------------------------------------------ATAGCTGACAAGCTCGTTCA------------------------------------------------
8 J20 370251 x1    100.0%  ------------------------------------------------------------------------------------CTGACAAGCTCGTTCACATA--------------------------------------------
```

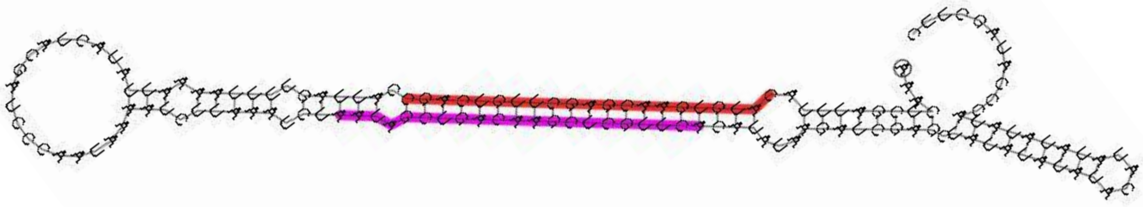

Jcu_nMIR012

```
1 Jcu_nmiR012       100.0%  ATTGATTCTGGCCAAGGCCCTACAAGGTGTATGCTAGAAGAAACAAAGGAGCTGTAATTGAATGTGTGAGTATTGGGGACCAAGCAAAGGAATACACGTCACCAAGTTAGCTATCAAATCATCACCCAGCAGTATATAAGGAA
2 J22_2034591_x1    100.0%  -----------------------------------------------------------------------------------------------------------------------------------------------
3 J22_1966484_x1    100.0%  -----------------------------------------------------------------------------------------------------------------------------------------------
4 J20_296601_x1     100.0%  -----------------------------------------------------------------------------------------------------------------------------------------------
5 J21_2562169_x1    100.0%  -----------------------------------------------------------------------------------------------------------------------------------------------
6 J21_2013458_x16   100.0%  -----------------------------------------------------------------------------------------------------------------------------------------------
7 J21_2012834_x16   100.0%  -----------------CCCTACAAGGTGTATGCTAGA---------------------------------------------------------------------------------------------------------
8 J22_1891920_x1    100.0%  -----------------CCCTACAAGGTGTATGCTAGAA--------------------------------------------------------------------------------------------------------

GCAAAGAGAATGATGAGGGCATCTTTGGGAATTCAATTATTTTGGGCAGAGGAATCTGCATGGGCTTGGCCTGGAGACTGTATCAGCCATTTATCTTCTTCTAGTATAAACCTTGTAGGGCCTTGGGCCGGAATCAATTCTATCA
--------------------------------------------------------------------------------------------------------------------------TTGGGCCGGAATCAATTCTATC-
---------------------------------------------------------------------------------------------------------------------GGGCCTTGGGCCGGAATCAATT------
----------------------------------------------------------------------------------------------------------------------GGCCTTGGGCCGGAATCAAT-------
----------------------------------------------------------------------------------------------------CTAGTATAAACCTTGTAGGGC------------------------
------------------------------------------------------------------------------------------------------TAGTATAAACCTTGTAGGGCC----------------------
------------------------------------------------------------------------------------------------------------------------------------------------
-------------------------------------------------------------------------------------------------------------------------------------------------
```

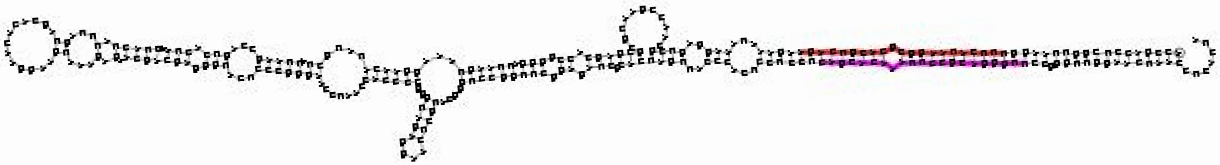

Jcu_nMIR013

```
 1 Jcu_nmiR013      100.0%  AAGAGGAAAAGAGATGGAATGGAGGAAGTTTTTGGACATGGGAGGATTTGCAAGAAAGATATTCTAAATCTTATTTCTGTTCTTGCCAATCCCTCCCATGCCAATGATTTCCTCTCTTCTCTATCCCTTTCCCTTCCTTCTC
 4 J24_5103366_x1   100.0%  -------------------------------TTGGACATGGGAGGATTTGCAAGA---------------------------------------------------------------------------------------
 5 J25_99258_x2     100.0%  -------------------------------TTGGACATGGGAGGATTTGCAAGAA--------------------------------------------------------------------------------------
 6 J24_2512829_x2   100.0%  --------------------------------TGGACATGGGAGGATTTGCAAGAA--------------------------------------------------------------------------------------
 7 J23_464792_x4    100.0%  --------------------------------TGGACATGGGAGGATTTGCAAGA---------------------------------------------------------------------------------------
 8 J24_3584151_x1   100.0%  ---------------------------------GGACATGGGAGGATTTGCAAGAAA-------------------------------------------------------------------------------------
 9 J21_2300580_x1   100.0%  ----------------------------------GACATGGGAGGATTTGCAAGA---------------------------------------------------------------------------------------
10 J22_1539146_x64  100.0%  --------------------------------TGGACATGGGAGGATTTGCAAG----------------------------------------------------------------------------------------
11 J21_1958127_x29  100.0%  -----------------------------------ACATGGGAGGATTTGCAAGAA--------------------------------------------------------------------------------------
12 J22_1495001_x94  100.0%  -----------------------------------ACATGGGAGGATTTGCAAGAAA-------------------------------------------------------------------------------------
13 J21_2046276_x11  100.0%  ---------------------------------GGACATGGGAGGATTTGCAAG----------------------------------------------------------------------------------------
14 J20_348455_x1    100.0%  ---------------------------------GGACATGGGAGGATTTGCAA-----------------------------------------------------------------------------------------
15 J21_1983632_x22  100.0%  ------------------------------------CATGGGAGGATTTGCAAGAAA-------------------------------------------------------------------------------------
16 J20_163237_x214  100.0%  -----------------------------------ACATGGGAGGATTTGCAAGA---------------------------------------------------------------------------------------
17 J21_1894725_x56  100.0%  -------------------------------TTGGACATGGGAGGATTTGCA------------------------------------------------------------------------------------------
18 J19_194870_x2    100.0%  ------------------------------------CATGGGAGGATTTGCAAGA---------------------------------------------------------------------------------------
19 J20_331047_x1    100.0%  -------------------------------TTGGACATGGGAGGATTTGC-------------------------------------------------------------------------------------------
20 J19_200573_x1    100.0%  -------------------------------------ATGGGAGGATTTGCAAGAA--------------------------------------------------------------------------------------
21 J20_258920_x1    100.0%  --------------------------------TGGACATGGGAGGATTTGCA------------------------------------------------------------------------------------------
22 J19_182371_x4    100.0%  -----------------------------------ACATGGGAGGATTTGCAAG----------------------------------------------------------------------------------------
23 J19_194314_x2    100.0%  --------------------------------TGGACATGGGAGGATTTGC-------------------------------------------------------------------------------------------
24 J19_216042_x1    100.0%  --------------------------------------TGGGAGGATTTGCAAGAAA-------------------------------------------------------------------------------------
25 J18_188421_x1    100.0%  -------------------------------TTGGACATGGGAGGATTT---------------------------------------------------------------------------------------------
26 J20_373860_x1    100.0%  ----------------------------------------GGAGGATTTGCAAGAAAGAT----------------------------------------------------------------------------------
27 J22_1875492_x2   100.0%  ----------------------AGGAAGTTTTTGGACATGGGAG--------------------------------------------------------------------------------------------------
28 J21_2063297_x9   100.0%  -----------------------GGAAGTTTTTGGACATGGGAG--------------------------------------------------------------------------------------------------
29 J22_1637299_x27  100.0%  -----------------------GGAAGTTTTTGGACATGGGAGG-------------------------------------------------------------------------------------------------
30 J21_2491823_x1   100.0%  ------------------------GAAGTTTTTGGACATGGGAGG-------------------------------------------------------------------------------------------------
31 J20_234248_x2    100.0%  -----------------------GGAAGTTTTTGGACATGGGA---------------------------------------------------------------------------------------------------
32 J20_225090_x3    100.0%  ------------GATGGAATGGAGGAAGTTTT--------------------------------------------------------------------------------------------------------------
33 J24_2564883_x2   100.0%  ------------GATGGAATGGAGGAAGTTTTTGGA----------------------------------------------------------------------------------------------------------
34 J25_152384_x1    100.0%  -------------ATGGAATGGAGGAAGTTTTTGGACA--------------------------------------------------------------------------------------------------------
35 J24_2580341_x2   100.0%  --------------TGGAATGGAGGAAGTTTTTGGACA--------------------------------------------------------------------------------------------------------
36 J20_327468_x1    100.0%  -------------ATGGAATGGAGGAAGTTTTT-------------------------------------------------------------------------------------------------------------
37 J19_229153_x1    100.0%  -------------ATGGAATGGAGGAAGTTTT--------------------------------------------------------------------------------------------------------------
38 J21_2155176_x3   100.0%  --------------------------------------------------------------------------------TCTTGCCAATCCCTCCCATGC-----------------------------------------
39 J22_1509420_x83  100.0%  --------------------------------------------------------------------------------TCTTGCCAATCCCTCCCATGCC----------------------------------------
40 J22_1795684_x5   100.0%  ---------------------------------------------------------------------------------CTTGCCAATCCCTCCCATGCCA---------------------------------------
41 J23_592928_x2    100.0%  ---------------------------------------------------------------------------------CTTGCCAATCCCTCCCATGCCAA--------------------------------------
42 J22_1407177_x204 100.0%  ----------------------------------------------------------------------------------TTGCCAATCCCTCCCATGCCAA--------------------------------------
43 J21_2013698_x16  100.0%  ----------------------------------------------------------------------------------TTGCCAATCCCTCCCATGCCA---------------------------------------
44 J20_359283_x1    100.0%  ---------------------------------------------------------------------------------CTTGCCAATCCCTCCCATGC-----------------------------------------
45 J21_2070239_x8   100.0%  -----------------------------------------------------------------------------------TGCCAATCCCTCCCATGCCAA--------------------------------------
46 J22_1783104_x5   100.0%  -----------------------------------------------------------------------------------TGCCAATCCCTCCCATGCCAAT-------------------------------------
47 J20_221259_x4    100.0%  ----------------------------------------------------------------------------------TTGCCAATCCCTCCCATGCC----------------------------------------
48 J19_187554_x2    100.0%  ----------------------------------------------------------------------------------TTGCCAATCCCTCCCATGC-----------------------------------------
49 J19_289868_x1    100.0%  -------------------------------------------------------------------------------------CCAATCCCTCCCATGCCAA--------------------------------------
50 J21_2132127_x4   100.0%  -------------------------------------------------------------------------------------CCAATCCCTCCCATGCCAATG------------------------------------
51 J22_1865420_x2   100.0%  -------------------------------------------------------------------------------------CCAATCCCTCCCATGCCAATGA-----------------------------------
52 J18_164830_x1    100.0%  -----------------------------------------------------------------------------------TGCCAATCCCTCCCATGC-----------------------------------------
53 J20_233478_x2    100.0%  ---------------------------------------------------------------------------------------------TCCCATGCCAATGATTTCCT-----------------------------
54 J21_1997538_x19  100.0%  ---------------------------------------------------------------------------------------------TCCCATGCCAATGATTTCCTC----------------------------
```

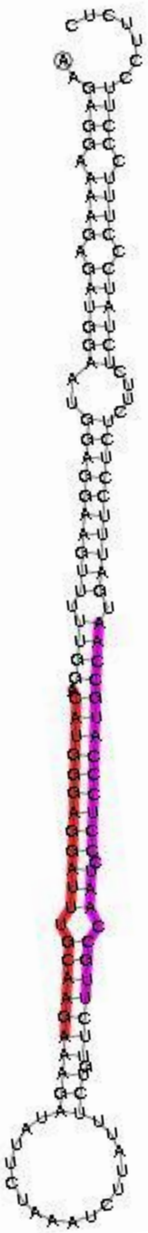

Jcu_nMIR014

```
 1 Jcu_nmiR014      100.0%  -----------GGCCTCGAACAAGTTGCTACATTTTTTTAACTTATTACAGTTACTTTTTCAAAATGAATTGGTAAAAGAAACTATGCATTTTTAATATTTATTTTTTATTCAATTTGTTGTGAATTTTAC
 2 J25_72643_x4     100.0%  -----------GGCCTCGAACAAGTTGCTACATTTT------------------------------------------------------------------------------------------
 3 J24_2901727_x2   100.0%  -----------GGCCTCGAACAAGTTGCTACATTT-------------------------------------------------------------------------------------------
 4 J23_662970_x1    100.0%  -----------GGCCTCGAACAAGTTGCTACATT--------------------------------------------------------------------------------------------
 5 J23_505343_x3    100.0%  -------------CCTCGAACAAGTTGCTACATTTT------------------------------------------------------------------------------------------
 6 J22_1881296_x2   100.0%  -------------CCTCGAACAAGTTGCTACATTT-------------------------------------------------------------------------------------------
 7 J22_1731653_x10  100.0%  --------------CTCGAACAAGTTGCTACATTTT------------------------------------------------------------------------------------------
 8 J21_1950501_x31  100.0%  ---------------TCGAACAAGTTGCTACATTTT------------------------------------------------------------------------------------------
10 J21_2113311_x5   100.0%  --------------CTCGAACAAGTTGCTACATTT-------------------------------------------------------------------------------------------
11 J20_295980_x1    100.0%  ----------------CGAACAAGTTGCTACATTTT------------------------------------------------------------------------------------------
12 J20_207763_x11   100.0%  ---------------TCGAACAAGTTGCTACATTT-------------------------------------------------------------------------------------------
13 J19_280222_x1    100.0%  -----------------GAACAAGTTGCTACATTTT------------------------------------------------------------------------------------------
14 J18 178145 x1    100.0%  -----------------GAACAAGTTGCTACATTT-------------------------------------------------------------------------------------------
```

```
AGTGTCCTTTCAAGTTTAATTGGGATTATTAGCTGATGAATCAACCTAGTTCTGTTCAAAATTCATGTTGGAAATTAAGTTCTAGAGCTTCAAATATTGATGGTTTTTTGTATAAGTTCCGGTGATTGTTGGATGTTAATAGAAAGTGTTAGGCGGCTTTTTTAGGGTAGATACTGAGTGCAGTTATTAAAATTGCTTGTCTTGGA
------------------------------------------------------------------------------------------------------------------------------------------------------------------------------------------------------
------------------------------------------------------------------------------------------------------------------------------------------------------------------------------------------------------
------------------------------------------------------------------------------------------------------------------------------------------------------------------------------------------------------
------------------------------------------------------------------------------------------------------------------------------------------------------------------------------------------------------
------------------------------------------------------------------------------------------------------------------------------------------------------------------------------------------------------
------------------------------------------------------------------------------------------------------------------------------------------------------------------------------------------------------
------------------------------------------------------------------------------------------------------------------------------------------------------------------------------------------------------
------------------------------------------------------------------------------------------------------------------------------------------------------------------------------------------------------
------------------------------------------------------------------------------------------------------------------------------------------------------------------------------------------------------
------------------------------------------------------------------------------------------------------------------------------------------------------------------------------------------------------
------------------------------------------------------------------------------------------------------------------------------------------------------------------------------------------------------
------------------------------------------------------------------------------------------------------------------------------------------------------------------------------------------------------
```

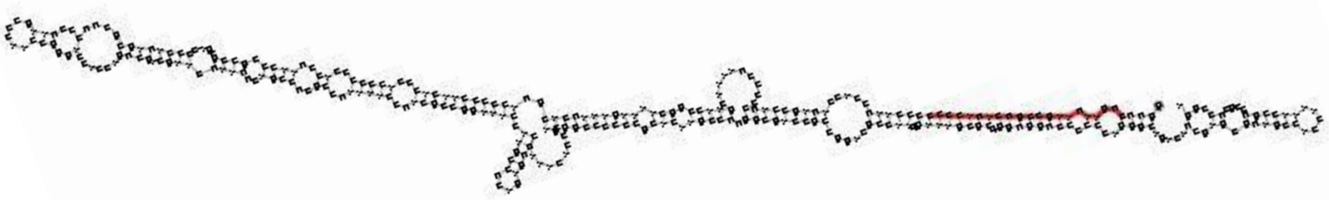

Jcu_nMIR015

```
 1 Jcu_nmiR015        100.0%  GAAGCGTAGGCGAGGGATTTTGCTTCGTATCAGAAAGTAAAGGGAGATAGAATTCATTGGAAGCTTTTGGCATGGGCGATATGGGCAAGATGAAAATCTCTGTGAATCATCTTACCCACACCCCCCATACCGATGGTTTTCAATAATTCATCCCTTTCCTTCTCTTTTCACTTACCGTATCCGA
 2 J24_640006_x13     100.0%  -------------------------------------------------------------------------------------------------------------TCTTACCCACACCCCCCATACCGA---------------------------------------------------
 3 J23_994672_x1      100.0%  --------------------------------------------------------------------------------------------------------------CTTACCCACACCCCCCATACCGA---------------------------------------------------
 4 J23_583330_x2      100.0%  ---------------------------------------------------------------------------------------------------------------TTACCCACACCCCCCATACCGAT--------------------------------------------------
 5 J22_1910076_x1     100.0%  -------------------------------------------------------------------------------------------------------------TCTTACCCACACCCCCCATACC-----------------------------------------------------
 6 J22_1257953_x1635  100.0%  ---------------------------------------------------------------------------------------------------------------TTACCCACACCCCCCATACCGA---------------------------------------------------
 7 J21_2519832_x1     100.0%  -------------------------------------------------------------------------------------------------------------TCTTACCCACACCCCCCATAC------------------------------------------------------
 8 J21_1733438_x237   100.0%  ----------------------------------------------------------------------------------------------------------------TACCCACACCCCCCATACCGA---------------------------------------------------
 9 J21_2427790_x1     100.0%  ---------------------------------------------------------------------------------------------------------------TTACCCACACCCCCCATACCG----------------------------------------------------
10 J20_296679_x1      100.0%  -----------------------------------------------------------------------------------------------------------------ACCCACACCCCCCATACCGA---------------------------------------------------
11 J20_222539_x4      100.0%  ---------------------------------------------------------------------------------------------------------------TTACCCACACCCCCCATACC-----------------------------------------------------
12 J19_269574_x1      100.0%  ---------------------------------------------------------------------------------------------------------------TTACCCACACCCCCCATAC------------------------------------------------------
13 J18_150285_x2      100.0%  ---------------------------------------------------------------------------------------------------------------TTACCCACACCCCCCATA-------------------------------------------------------
14 J18_169283_x1      100.0%  -------------------------------------------------------------------------------------------------------------------CCACACCCCCCATACCGA---------------------------------------------------
15 J21_2508154_x1     100.0%  -----------------------------------------------------------------------------------------------------------------------------CATACCGATGGTTTTCAATAA--------------------------------------
18 J18_160042_x1      100.0%  --------------------------------------------------------------------GGCATGGGCGATATGGGC--------------------------------------------------------------------------------------------------
19 J19_184544_x3      100.0%  --------------------------------------------------------------------GGCATGGGCGATATGGGCA-------------------------------------------------------------------------------------------------
20 J21_2215795_x2     100.0%  --------------------------------------------------------------------GGCATGGGCGATATGGGCAAG-----------------------------------------------------------------------------------------------
21 J22_1580795_x44    100.0%  --------------------------------------------------------------------GGCATGGGCGATATGGGCAAGA----------------------------------------------------------------------------------------------
22 J24_849914_x10     100.0%  --------------------------------------------------------------------GGCATGGGCGATATGGGCAAGATG--------------------------------------------------------------------------------------------
23 J20_362336_x1      100.0%  ------------------------------------------------------------------------TGGGCGATATGGGCAAGATG--------------------------------------------------------------------------------------------
24 J21_2016920_x15    100.0%  ------------------------------------------------------------------------TGGGCGATATGGGCAAGATGA-------------------------------------------------------------------------------------------
```

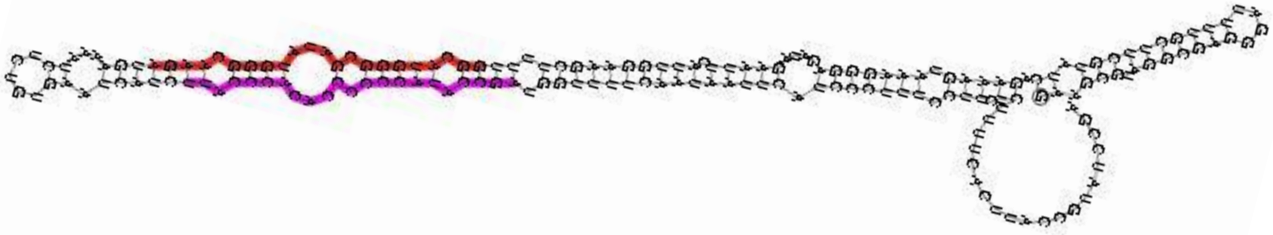

Jcu_nMIR016

```
 1 Jcu_nmiR016        100.0%  AAGAAGGGAAAGGTTTTGGACGCAACTTGGAGTGGCACCATTAACACTTGGATAGGGTGGGAGTAAATGGTGCCACGCTGAGTGCGTCTAAAATACTTGCCCTTTGAATA
 2 J25_263445_x1      100.0%  ------------GTTTTGGACGCAACTTGGAGTGGCA------------------------------------------------------------------------
 3 J25_151515_x1      100.0%  --------------TTTGGACGCAACTTGGAGTGGCACC----------------------------------------------------------------------
 4 J22_1848618_x2     100.0%  -------------TTTTGGACGCAACTTGGAGTGG--------------------------------------------------------------------------
 5 J22_1790169_x5     100.0%  --------------TTTGGACGCAACTTGGAGTGGC-------------------------------------------------------------------------
 6 J19_179638_x6      100.0%  --------------------CGCAACTTGGAGTGGCACC----------------------------------------------------------------------
 7 J22_1306546_x680   100.0%  --------------------CGCAACTTGGAGTGGCACCATT-------------------------------------------------------------------
 8 J21_2084322_x7     100.0%  ---------------------GCAACTTGGAGTGGCACCATT-------------------------------------------------------------------
 9 J20_337129_x1      100.0%  ------------------------------------------------------------------TGGTGCCACGCTGAGTGCGT-----------------------
10 J21_1873402_x70    100.0%  ------------------------------------------------------------------TGGTGCCACGCTGAGTGCGTC----------------------
11 J18_194143_x1      100.0%  ---------------------------------------------------------------------TGCCACGCTGAGTGCGTC----------------------
```

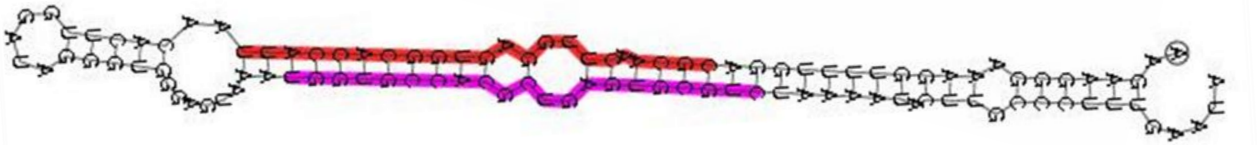

Supplement: Figure S3 — Predicted secondary structures of novel miRNA precursors in J. curcas . The locations and the expression of small RNAs mapped onto these precursors are shown here. The sequences of miRNA candidates located in the 5p and 3p arms are labeled in red and purple, respectively. Values on the left side of the miRNA sequence represent miRNA length (Jn) and read counts (x n) in the mature seed library. (PDF) [file pone.0083727.s003.pdf]
